# Low and Variable Correlation Between Reaction Time Costs and Accuracy Costs Explained by Accumulation Models: Meta-Analysis and Simulations

**DOI:** 10.1037/bul0000164

**Published:** 2018-09-27

**Authors:** Craig Hedge, Georgina Powell, Aline Bompas, Solveiga Vivian-Griffiths, Petroc Sumner

**Affiliations:** 1 School of Psychology, Cardiff University

**Keywords:** Reaction time costs, error costs, individual differences, accumulation models, sequential sampling models

## Abstract

The underpinning assumption of much research on cognitive individual differences (or group differences) is that task performance indexes cognitive ability in that domain. In many tasks performance is measured by differences (costs) between conditions, which are widely assumed to index a psychological process of interest rather than extraneous factors such as speed–accuracy trade-offs (e.g., Stroop, implicit association task, lexical decision, antisaccade, Simon, Navon, flanker, and task switching). Relatedly, reaction time (RT) costs or error costs are interpreted similarly and used interchangeably in the literature. All of this assumes a strong correlation between RT-costs and error-costs from the same psychological effect. We conducted a meta-analysis to test this, with 114 effects across a range of well-known tasks. Counterintuitively, we found a general pattern of weak, and often no, association between RT and error costs (mean *r* = .17, range −.45 to .78). This general problem is accounted for by the theoretical framework of evidence accumulation models, which capture individual differences in (at least) 2 distinct ways. Differences affecting accumulation rate produce positive correlation. But this is cancelled out if individuals also differ in response threshold, which produces negative correlations. In the models, subtractions between conditions do not isolate processing costs from caution. To demonstrate the explanatory power of synthesizing the traditional subtraction method within a broader decision model framework, we confirm 2 predictions with new data. Thus, using error costs or RT costs is more than a pragmatic choice; the decision carries theoretical consequence that can be understood through the accumulation model framework.

## Public Significance Statement

Our meta-analysis reveals that RT costs and error costs from the same psychological effects do not correlate, contrary to widespread assumption. This is explained if people vary in both caution and cognitive abilities. We demonstrate this by simulating data from 4 models in the broad family of evidence accumulation models. Individual differences in behavior should not be assumed to solely reflect individual differences in ability in a cognitive domain.

Sixty years ago prominent psychologists worried about an inevitable parting of ways between two disciplines of psychology, as eloquently highlighted by Cronbach (1957):

No man can be acquainted with all of psychology today. . . [There is] plentiful evidence that psychology is going places. But Whither? . . . The personality, social and child psychologists went one way; the perception and learning psychologists went the other; and the country between turned into desert. (pp. 671–673)

The different sides across the desert followed different approaches: on one side, differences between individuals were the very focus of study, while on the experimental side “individual variation is cast into that outer darkness known as ‘error variance’” (Cronbach, 1957, p. 674). It might therefore please Cronbach that experimental tasks are now increasingly employed in the study of individual differences. This bridge is occurring across several fields, for example in cognitive neuroscience in the search for neural correlates of performance (e.g., Kanai & Rees, 2011; Sumner, Edden, Bompas, Evans, & Singh, 2010), in mental health research in the search for cognitive predictors for disease or endophenotypes of genetic risk factors (Carter & Barch, 2007), or in the search for cognitive mechanisms underlying personality dimensions such as impulsivity (Cyders & Coskunpinar, 2011, 2012; Sharma, Kohl, Morgan, & Clark, 2013; Sharma, Markon, & Clark, 2014). However, the interpretation of individual variation in cognitive tasks turns out to be less straightforward than is often assumed; counterintuitive phenomena occur in the “outer darkness.”

One of the cornerstones of experimental psychology is the subtraction method (Donders, 1969), in which performance in one experimental condition is subtracted from another condition involving additional processes, to calculate a performance “cost” or “effect” assumed to largely isolate the processes of interest from more general factors such as arousal or speed–accuracy trade-offs (Broota, 1989, p. 396; Gravetter & Forzano, 2015, p. 266; Greenwald, 1976, p. 315). Examples include well-known effects in widely used tasks across multiple domains, such as the Eriksen flanker effect (Eriksen & Eriksen, 1974), Stroop effect (Stroop, 1935), Simon effect (Simon & Wolf, 1967), antisaccade cost (Hallett, 1978), remote distractor effect (Walker, Kentridge, & Findlay, 1995), SNARC effect (SNARC; Dehaene, Dupoux, & Mehler, 1990), Navon global and local effects (Navon, 1977), task-switching cost (Jersild, 1927; Monsell, 2003), implicit association effect (IAT; Greenwald, McGhee, & Schwartz, 1998), attentional effects (Fan, McCandliss, Sommer, Raz, & Posner, 2002), and lexical decision costs (Meyer & Schvaneveldt, 1971).

These kinds of performance costs typically occur in both reaction times (RT) and error rates, and terms such as “Stroop effect,” “implicit association effect,” “attentional cost,” or “switch cost” can refer to either RT costs or error costs interchangeably. As such researchers tend to assume that both reflect the same underlying mechanisms, and whether to use error or RT costs is seen as a pragmatic choice rather than one with theoretical consequence. For some paradigms it is traditional to focus on one measure, for example, RT costs in task switching or the IAT, but it is nevertheless expected that effects of interest will also be reflected in error rates (Draheim, Hicks, & Engle, 2016; Nosek, Bar-Anan, Sriram, Axt, & Greenwald, 2014).

When moving from group effects to individual differences or group differences, the theoretical basis of many conclusions depends on the assumption that differences in performance costs reflect variance in processing ability in that cognitive domain. More able participants should have smaller costs in both RT and errors, once speed-accuracy trades-offs are subtracted out. In other words, RT and error costs should correlate. Empirical studies and meta-analyses tend to draw upon both error costs and RT costs and use either to support the same conclusions. To take just two examples, if we dissect a recent meta-analysis of response control in autism spectrum disorders, which included 16 data sets from flanker, Simon, and Stroop tasks (Geurts, van den Bergh, & Ruzzano, 2014), we find five showed effects for RT costs while three showed effects for error costs (see Supplementary Material A). Similarly, in a meta-analysis of 12 studies examining flanker and Simon effects in children with attention-deficit/hyperactivity disorder (Mullane, Corkum, Klein, & McLaughlin, 2009), three studies observed larger RT costs and two observed increased error costs. None of the data sets in either meta-analysis showed effects for RT costs and error costs simultaneously, hinting that the assumption of equivalence might not hold.

Using performance costs (subtraction between conditions) has been so successful and ubiquitous in experimental research, that when moving to study individual differences, it is rarely questioned whether individual differences in RT costs or error costs primarily reflect processing ability, or whether they might in fact reflect other factors such as differences in strategy. When not using costs, but rather absolute accuracy or RT in tasks, it is appreciated that strategy, cautiousness, and other factors may contaminate individual differences. For example, in numeracy tasks it has been illustrated how absent correlations between tasks can be explained by dissociating information processing and caution using a quantitative model (Ratcliff, Thompson, & McKoon, 2015). For most researchers, such complications with absolute RT or accuracy are exactly the reason why they subtract between conditions—the resulting cost score is supposed to be immune from contamination.

However, across the literature are many hints implying all is not well with the assumptions underlying correlational research with cognitive performance costs. Draheim, Hicks, and Engle (2016) have recently questioned why RT task switch costs show inconsistent or no relationship with measures of working memory capacity even though theorists generally agree that working memory is implicated in task switching (cf. Monsell, 2003). Similarly, it is often assumed that different response conflict tasks tap common underlying control mechanisms (cf. Friedman & Miyake, 2004; Miyake et al., 2000), but correlations between tasks are often low or absent (Aichert et al., 2012; Fan, Flombaum, McCandliss, Thomas, & Posner, 2003; Khng & Lee, 2014; Scheres et al., 2004; Wager et al., 2005). For the IAT task, recent meta-analyses of the extent to which attitudes or behavior can be predicted by task scores have reached mixed conclusions (Greenwald, Poehlman, Uhlmann, & Banaji, 2009; Oswald, Mitchell, Blanton, Jaccard, & Tetlock, 2013; though see, Greenwald, Banaji, & Nosek, 2015). The absence of theoretically predicted relationships between supposedly related tasks is a challenge for these theories, and has led researchers to question the selection of measures.

The contamination of RT costs by processes not specific to the domain of interest has been discussed previously (Faust, Balota, Spieler, & Ferraro, 1999; Miller & Ulrich, 2013). Miller and Ulrich (2013) propose a broad stage-based framework for individual differences in RT (IDRT), wherein RT arises from the sum of processing durations across perceptual input, response selection and motor output stages. Although this framework treats only RT and is agnostic about the mechanisms within these stages and the sources of general and specific variance in terms of psychological process, Miller and Ulrich (2013) highlight two important things for our discussion: RT costs are not a pure index of individual differences in the theoretical mechanisms that they are frequently used to represent; and if variance between individuals arises from both task-specific and general processing factors, it can become difficult to interpret correlations.

In order to obtain a more complete representation of behavioral performance, some authors have used composite measures of RT and accuracy (Draheim et al., 2016; Hughes, Linck, Bowles, Koeth, & Bunting, 2014; Khng & Lee, 2014; Mullane et al., 2009; Stahl et al., 2014; Townsend & Ashby, 1978, 1983). However, such methods still generally assume that RT costs and error costs reflect the same mechanisms—at least in part—and thus will positively correlate. In contrast, absent correlation between RT costs and error costs within the same Stroop task led Kane and Engle (2003) to suggest the two measures actually reflect different mechanisms (conflict resolution and goal maintenance).

## Overview of the Article

In Part 1 of this article, we perform a meta-analysis to test the widespread theoretical assumption underpinning the use of performance costs as indexes of ability in specific cognitive domains. This assumption predicts a positive correlation between performance measures—error costs and RT costs—within the same task. This assumed correlation supplies an implicit justification to choose either measure on pragmatic grounds without theoretical consequence (or to combine them into a single metric). We test the correlation for 114 experimental effects taken from 43 different studies, encompassing 13 prominent paradigms across experimental psychology, using both new data and reanalysis of previously published data from many labs (originally addressing many different questions). To anticipate, we find little or no correlation in the majority of cases; for example, an individual’s Stroop effect measured by errors is clearly not interchangeable with their Stroop effect measured by RT, and likewise for nearly all the other common effects we analyze.

Should we be alarmed by this? From most theoretical standpoints, this general pattern seems surprising and potentially undermines the conclusions of many studies, reviews and meta-analyses. But from one family of theoretical perspectives is it not alarming, as we illustrate in Part 2 of the article using four different models (Bompas & Sumner, 2011; Brown & Heathcote, 2008; Ratcliff & Rouder, 1998; Ulrich, Schröter, Leuthold, & Birngruber, 2015) drawn as exemplars from a wider family of models that employ an evidence accumulation framework (Bogacz, Usher, Zhang, & McClelland, 2007; Carpenter & Williams, 1995; Hübner, Steinhauser, & Lehle, 2010; Logan, Cowan, & Davis, 1984; Teodorescu & Usher, 2013; Usher & McClelland, 2001; White, Ratcliff, & Starns, 2011). It turns out that within this framework, absent or inconsistent correlation between RT costs and error costs should be expected. This theoretical prediction emerges from the same model features that explain why absolute accuracy and RT did not correlate in numeracy tasks (Ratcliff et al., 2015).

The models capture individual differences in (at least) two distinct ways. The first corresponds to differences in accumulation rates (processing or selection efficiency). When individuals vary only in their selection efficiency, this produces a positive correlation between RT costs and error costs, as commonly assumed. The second corresponds to response threshold (caution), where differences would produce a negative correlation between error costs and RT costs. Note that because we are dealing with costs calculated through subtraction, not absolute RT and error rates, this negative correlation is not a simple speed–accuracy trade-off. However, a key theoretical consequence of these models is that threshold and processing efficiency interact, and a subtraction between conditions does not control for caution differences between individuals.

If participants vary in both accumulation rate (selection efficiency) and threshold, then an overall correlation between error and RT costs is not expected, despite both being outcomes of the same decision and control mechanisms. We illustrate that this is not a feature of any specific model, but a property the family shares. Thus, the framework of accumulator models appears fruitful for understanding individual differences in performance on choice decision tasks.

In Part 3 of the article we test with new data two predictions arising from the modeling framework. First, reducing variance in response caution by emphasizing speed (cf. Ratcliff et al., 2015) should mean the correlation between RT costs and error costs becomes more positive. We test this with meta-analysis of recent unpublished studies using a speed–accuracy trade-off design. Second, reducing the opportunity for participants to adopt strategic caution differences by randomly intermixing trial conditions within blocks should also lead to more positive correlations, compared with when trial conditions are performed in separate blocks, which allows more variability in strategy. We test this with new data directly comparing the same task with intermixed or blocked conditions. Both of these predictions were corroborated, leading us to accept the accumulation model family as a suitable theoretical framework for understanding individual differences in performance costs in cognitive tasks.

## Part 1: No Consistent Correlation Between RT Costs and Error Costs in Cognitive Tasks

### Method

#### Search strategy

We identified a list of widely used and cited speeded choice tasks for which performance can be measured with either RT costs or error costs (i.e., a subtraction between two types of condition), and for which we were able to access at least one suitable dataset from open science resources, our own studies, or from colleagues.

**Figure 1 fig1:**
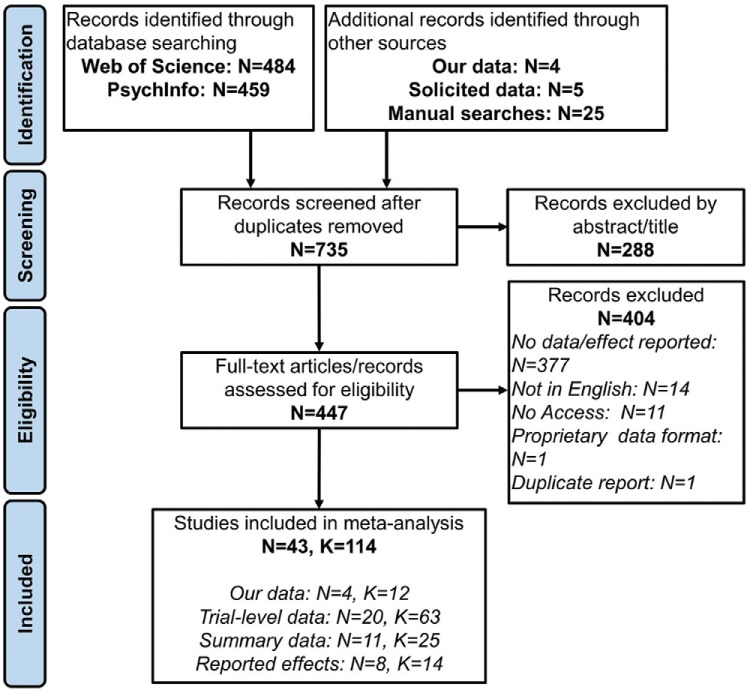
PRISMA flow diagram illustrating our process for identifying eligible articles and datasets. N refers to records (articles or records on data repositories), K refers to correlations identified. Manual searches refers to records obtained through reference lists, Google, and manually searching data repositories (e.g. OSF.io).

We used the following strategies to search for relevant literature: (a) PsycINFO and Web of Science. Our search terms were any of the task names: “flanker,” “Stroop,” “Simon,” “antisaccade,” “remote distractor,” “snarc,” “Navon,” “task-switch,” “implicit association test,” “attention network test,” and “lexical decision;” in combination with any of the terms: “RT cost,” “RT cost,” “error cost,” “accuracy cost,” “latency cost,” and “cost.” We supplemented this search by manually searching Google and Google Scholar with the same terms, and scanning the reference lists of eligible articles. We included unpublished research dissertations in our search. (b) Then, we searched for additional data sets from which RT costs and error costs could be calculated. We searched within the Open Science Framework (https://osf.io/) for each task by name, as well as searching Google for “(task name) dataset.” We required data sets to have an associated article or preprint, in order to identify necessary study information. (c) A further 12 correlations from eight different tasks were collected in our own lab. Six of these correlations come from a previously published article (Hedge, Powell, & Sumner, 2017), the others are unpublished data collected in part to address this question. The descriptions, and a figure summarizing the format of these tasks is included in Supplementary Material B. (d) Data from five studies was made available to us by colleagues. See Table 1 for sample sizes and trial numbers. See Supplementary Material C for additional details on how the data were extracted. See Figure 1 for our PRISMA flow diagram (Moher et al., 2009).[Table-anchor tbl1][Fig-anchor fig1]

Our inclusion criteria were that the study should either report the correlation between the RT cost and error cost, or the data should be made available such that we could calculate the correlation ourselves. For tasks that contained both a congruent and neutral condition, we use the congruent condition as a baseline, as we believed it to be more comparable with tasks that do not have a neutral condition (e.g., the IAT), and it is not always clear what constitutes a neutral stimulus (cf. Jonides & Mack, 1984; MacLeod, 1991). We did not exclude studies on the basis of age or clinical conditions, though eligible data sets from samples other than healthy adults were rare. Though we focused our search on particular paradigms that are widely used in individual differences research, eligible data sets often included other common manipulations and effects that we did not explicitly search for (e.g., comparing single task blocks with mixed task blocks in task-switching studies). We calculated the correlation between RT costs and error costs for these manipulations where appropriate. Our search produced 114 correlations in total (see Table 1).

Where the raw trial by trial data were available (k = 75, including our data), we applied a common preprocessing and outlier removal pipeline (see Data Analysis section below). Where we only obtained summary data for each participant (k = 25), the calculation of individual’s RT costs and error costs reflect the authors’ original outlier removal strategy. From each dataset, we extracted the sample size and trial number, which are reported in Table 1 along with each effect size. See Supplementary Table C1 for additional information for each study. Only five of these articles discussed the relationship between RT costs and error costs in any way, and we outline the content of such discussion in the Discussion section of Part 1 and the General Discussion section.

#### Data analysis

Where studies involved data collection over multiple sessions, we collapsed across sessions if possible. In some cases (e.g., Saunders, He, & Inzlicht, 2015) some participants did not have data for all sessions so we entered the sessions separately. We combined data from different experiments within the same article if the same protocol was replicated in multiple samples. The calculation of mean RTs excluded RTs below 100 ms (75 ms in eye movement tasks) and greater than three times each individual’s median absolute deviation from their median in each condition (Hampel, 1974; Leys, Ley, Klein, Bernard, & Licata, 2013). When only summary data were available, we removed individuals whose mean RTs were below 100 ms or their average accuracy across conditions was below 60%.

Effect sizes (Pearson’s *r* and Spearman’s rho) were calculated for the correlation between RT costs and error costs for each effect. Initially, we used Pearson’s *r* estimates in the meta-analysis because they were more common in existing reports. We then reran the analysis using Spearman’s rho estimates to minimize the impact of outliers in some data sets. In the conventional interpretation of these effect sizes, 0.1 is considered small, 0.3 is a medium effect size, and 0.5 is a large effect size (Cohen, 1988).

Meta-analyses were conducted using Hedges and colleagues’ method assuming a random-effects model (Hedges & Olkin, 1985; Hedges & Vevea, 1998). We assessed heterogeneity using the I^2^ statistic, which estimates the variance of the true effect sizes as a percentage of total variance (including sampling error). I^2^ values of 25%, 50%, and 75% are interpreted as low, moderate, and high levels of heterogeneity, respectively (Higgins, Thompson, Deeks, & Altman, 2003). We also conducted a metaregression analysis to assess whether effect size was moderated by the number of trials administered, which we centered on the mean. We did not include task/effect as a moderator due to the low number of data sets (sometimes one) obtained for some, though we conducted a post hoc sensitivity analysis to assess the impact of influential data points. All analyses were conducted using the metafor package (Viechtbauer, 2010) in R (R Core Team, 2016).

### Results and Discussion

Table 1 shows the correlations between RT costs and error costs observed for each experimental effect, grouped by their source, along with sample size and trial numbers.

**Figure 2 fig2:**
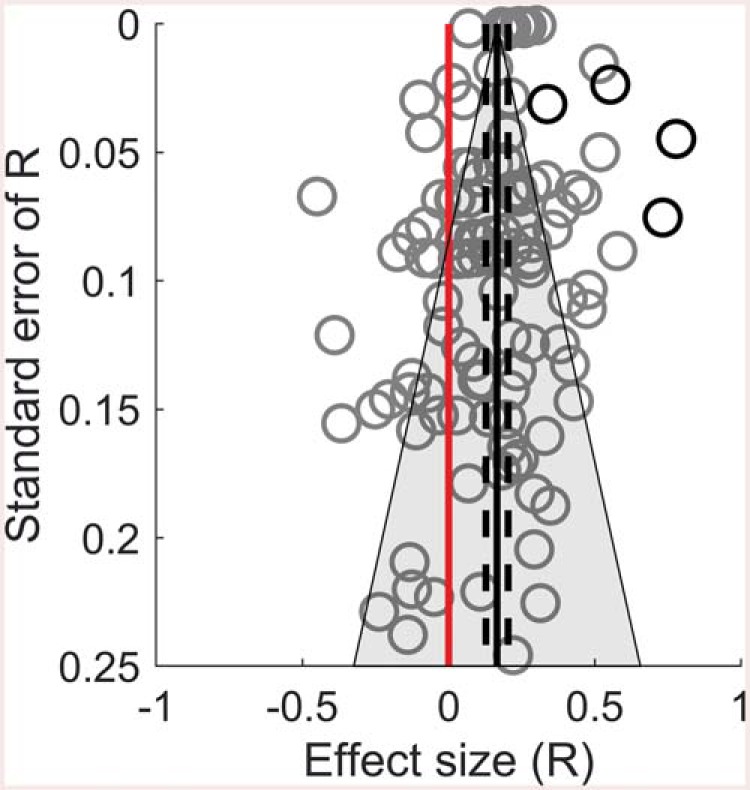
Funnel plot of observed effect sizes (Pearson’s r) for correlations between RT costs and error costs with associated standard errors. Larger values on the y-axis reflect larger sample sizes. Solid black line indicates weighted mean effect from a random effects model. Grey area indicates 95% confidence region. Dashed black lines show 95% confidence intervals of the mean effect estimated from a random-effects model. Red line indicates an effect size of zero. The lexical decision task effects are shown in black circles, all other tasks are shown in gray (see text for details).

The meta-analysis using Pearson’s *r* coefficients (k = 114) indicated that overall there was a small correlation between RT costs and error costs (*r* = .17, 95% CI [.13, .20], z = 8.54, *p* < .001), with a very high degree of between study heterogeneity (I^2^ = 99.9%). As can be seen in Figure 2, the observed Pearson’s *r* values ranged between −.45 and .78, with 79% of the absolute values falling below what is typically considered to be a moderate effect size (.3; Cohen, 1988). Rerunning the analysis using Spearman’s rho coefficients gave a slightly higher, but still small, estimate of the average effect (*r* = .19, 95% CI [.16, .23], z = 10.66, *p* < .001). Clearly individual differences in RT costs and error costs are not behaving as expected if they were interchangeable measures of the same cognitive processes.[Fig-anchor fig2]

#### Publication bias

To assess and control for potential biases, we conducted Egger’s test (Egger, Davey Smith, Schneider, & Minder, 1997), followed by a trim and fill analysis (Duval & Tweedie, 2000a, 2000b). Egger’s test assesses funnel plot asymmetry. When no bias exists, the effects observed in individual studies should be symmetrically distributed around the average effect. Alternatively, a tendency for studies with small sample sizes to show stronger effects is typically interpreted as an indication of publication bias, as small studies with nonsignificant effects are less likely to be published. A trim and fill analysis corrects for funnel plot asymmetry by simulating “missing” studies to make the funnel plot symmetrical. Egger’s test indicated a significant asymmetry (z = −2.62, *p* = .009). Inspection of Figure 2 indicates that this is not driven by a trend for smaller samples to show larger effects, rather, it is influenced by their relative *absence* (the middle- and lower-right section of the plot is relatively sparse). This is also influenced by the lexical-decision task data sets, which had relatively large positive correlations and sample sizes. The trim and fill analysis simulated studies with positive correlations to correct for this asymmetry, though the corrected estimate was still small (*r* = .25).

None of the published data sets we included were collected for the purpose of examining the correlation between RT costs and error costs, and it is unlikely that the size of that correlation formed any part of the publication decision process or the choice to make the data sets available (the correlation was not reported in most cases). Publication decisions in some studies would have depended on within-subject effects and hence favored low between-participants variance (and thus lower possibility for correlation, see Hedge et al., 2017; Miller & Ulrich, 2013; Paap & Sawi, 2016). However, the original research questions across the 114 data sets were neither predominantly within-subject (favoring low variance) nor correlational (favoring high variance) by nature, so the data sets should not be systematically biased toward either high or low between subject variability.

#### Trial number

Metaregression analysis indicated that the number of trials administered significantly predicted the size of the effect, with more trials associated with larger effects (b = .00004, z = 5.12, *p* < .001). However, examination of Table 1 indicates that this may be strongly influenced by the lexical decision studies, which are arguably outliers in their trial numbers, and also produced the highest correlations (see Discussion section below). To assess this, we reran the meta-analysis and moderator analysis with the four lexical decision studies excluded. In the remaining data sets (k = 110), the average effect was *r* = .15 (95% CI [.11, .18], z = 8.27, *p* < .001). A high degree of heterogeneity was again observed (I^2^ = 99.9%), though trial number no longer significantly predicted effect size (b = 0.00005, z = −.32, *p* = .75).

#### Specific task patterns

Though we did not conduct a formal moderator analysis for task, some trends are noteworthy from the examination of Table 1. The four lexical-decision task data sets show a range of moderate to strong positive correlations (R = .34 to .78). One possible reason for this is the large number of trials used in these studies, which may serve to minimize measurement error that would otherwise attenuate correlations (Hedge et al., 2017; Paap & Sawi, 2016). Alternatively, it may reflect different patterns of behavior produced by responses to words compared with nonwords. Most of the tasks we examine consist of a comparison between relatively easy trials and relatively hard trials (e.g., congruent vs. incongruent, task repetitions vs. task switches). The latter are expected to produce longer RTs and an increased error rate. This is often not the case in the lexical-decision task, where RTs are longer to nonwords but error rates are comparable or lower than for words (see Keuleers, Lacey, Rastle, & Brysbaert, 2012; Table 2). Keuleers, Lacey, Rastle, and Brysbaert (2012) suggest that high error rates to words may reflect other properties of the stimuli, for example, individuals may mistakenly identify low-frequency words as nonwords. The correlations we report may be strongly influenced by individual differences in factors that influence this behavior (for a recent discussion of nonword properties, see Yap, Sibley, Balota, Ratcliff, & Rueckl, 2015). However, it is important to note that studies utilizing the lexical-decision task for individual differences often employ controls on confounding stimulus properties such as frequency. We would not conclude on the basis of the strong correlations in Table 1 that the lexical-decision task is immune to the general issues raised by our analysis.

In the IDRT framework, Miller and Ulrich (2013) suggest that RT costs can be distinguished by whether they reflect common or opposing task-specific processes. In mental rotation, for example, rotating an object 180° draws upon the same mental process as rotating an object by 90°, but in a greater amount. In Stroop tasks, by contrast, reading automaticity is helpful in congruent conditions but unhelpful in incongruent conditions. RT costs derived from such opposing task-specific processes would be expected to have higher reliability, whereas RT costs derived from common-task specific processes would be expected to show stronger correlation with external measures. Most of the effects we include in our meta-analysis rely on opposing-processes, though lexical decision effects could be interpreted to rely on common processes. Models of lexical decision performance often specify a serial search of the mental lexicon (e.g., Coltheart, Rastle, Perry, Langdon, & Ziegler, 2001), where a word response is given if a matching entry is found, and a nonword response is given if no match is found by some point at which the search is terminated. Though Miller and Ulrich’s (2013) IDRT model does not address error costs, one could interpret the stronger correlations between RT costs and error costs in lexical decision as compatible with task-common processes. However, this extrapolation from Miller and Ulrich (2013) treats error costs as an “external measure” just like RT costs in different tasks.

The flanker task showed a wide range of correlations across 17 data sets (*r* = −.45 to .58). Notably, the two moderate negative correlations we observed in the flanker task were in Parkinson’s patients (*r* = −.39; Wylie et al., 2009) and older adults aged 65- to 80-years-old, respectively (*r* = −.45; Guye & Von Bastian, 2017). The latter correlation was influenced by an outlier, as indicated by the smaller Spearman’s correlation (ρ = −.08). Nevertheless, the same participants showed moderate positive correlations in the Simon (*r* = .38) and Stroop (*r* = .46) tasks in Guye and Von Bastian (2017) study, suggesting that negative correlations are not a general consequence of particular samples.

#### Reliability

How can the absence of a strong correlation between two indices of performance from the same task be reconciled with a (typically) robust effect on both metrics at a group level? One possibility is that the use of difference scores obscures a “true” underlying relationship. For statistical reasons, difference scores typically show less reliable individual differences than their component measures, and this will attenuate the correlations between them and other variables (Cronbach & Furby, 1970; Lord, 1956; Spearman, 1910). Previous authors have noted that this may be a reason why *different tasks* do not correlate as well as often expected (Draheim et al., 2016; Hedge et al., 2017; Khng & Lee, 2014; Miller & Ulrich, 2013; Paap & Sawi, 2016). The same issue would also affect the correlation between RT and error costs within tasks.

However, as a sole explanation, poor reliabilities do not account for the low magnitude of the correlations that we observe. Psychometricians have suggested formulae that use the reliabilities of two measures to “disattenuate” the observed correlation between them (Nunnally, 1970; Spearman, 1910). This procedure is intended to estimate what the relationship between two variables might be if not obscured by measurement error. For example, we previously found 3-week retest reliabilities ranging between .46 and .66 for Stroop and flanker effects (Hedge et al., 2017). Using these values would raise correlations of ∼.3 between error and RT costs to estimated disattenuated correlations of *r* ∼ .5. Similar levels of reliability are reported for other tasks (e.g., an average of .5 for the IAT; Lane, Banaji, Nosek, & Greenwald, 2007), and most of our measured correlations were below .3. Thus, most tasks would produce lower disattenuated estimates than .5. Although .5 is nominally considered to be a strong correlation between two separate factors (Cohen, 1988), 75% of the variance in one measure is not accounted for by the other and in this case we are correlating two measures supposed to reflect the same thing. Therefore, the assumption that RT and error costs are interchangeable measures is not justified even if reliability could be accounted for in this way.

### Interim Summary

Overall then, our analysis illustrates that widely used and robust effects in RTs and their corresponding effects in errors show inconsistent, and often very little, correlation. This challenges the theoretical framework in which we traditionally interpret and assess cognitive differences. For example, how does one interpret a deficit in response inhibition that specifically affects RT costs but not error costs? The production of two uncorrelated measures from each task also increases the likelihood of false positives if not statistically controlled (John, Loewenstein, & Prelec, 2012). This could be exacerbated by selective reporting in tasks where it is common to examine either RT or error costs without explicit justification for the choice.

Only five studies discussed the correlation between RT and error costs. Two studies (Cherkasoava et al., 2002; Manoach et al., 2002) report a negligible correlation in task switching in order to rule out the presence of a speed–accuracy trade-off. While the authors do not further interpret the absence of a positive correlation, the implication of their brief discussion is that they do not assume RT costs and error costs control for strategic changes. We return to the three other discussions for task switching (Draheim et al., 2016; Hughes et al., 2014) and the Stroop task (Kane & Engle, 2003) in the General Discussion section. First, in Part 2, we discuss how RTs and errors in cognitive tasks can be understood in the framework of evidence accumulation models.

## Part 2. Evidence Accumulation Models Explain Low Correlations

Evidence accumulation models are a method of analyzing and simulating RT and error rates in choice RT tasks, which have seen increasing use in recent years (for reviews and discussion, see Bogacz, Brown, Moehlis, Holmes, & Cohen, 2006; Donkin, Brown, Heathcote, & Wagenmakers, 2011; Forstmann, Ratcliff, & Wagenmakers, 2016; Forstmann & Wagenmakers, 2015; Forstmann, Wagenmakers, Eichele, Brown, & Serences, 2011; Ratcliff & Smith, 2004; Ratcliff, Smith, Brown, & McKoon, 2016; Teodorescu & Usher, 2013). The assumptions and architecture of these models vary, but all broadly assume an underlying process whereby evidence for the response alternatives is sampled sequentially over time, until a threshold is reached for one of the responses. A period of nondecision time is added to account for processes of stimulus encoding and motor initiation, but this part of the models is not relevant for our discussion here. These models are popular because their parameters can be linked to underlying cognitive and neurophysiological processes, and because they capture both error rates and RTs well in a unified framework.

For illustration, we focus on four models here: the drift-diffusion model (DDM; Ratcliff, 1978; Ratcliff & McKoon, 2008), the linear ballistic accumulator (LBA) model (Brown & Heathcote, 2008), the diffusion model for conflict tasks (DMC; Ulrich et al., 2015), and the approximately linear rise to threshold with ergodic rate (ALIGATER; Bompas & Sumner, 2011). There is ongoing debate about the precise nature of the modeled mechanisms and the assumptions each model makes in their implementation. This debate also extends to models not covered in detail here (for discussions, see Carpenter & Reddi, 2001; Donkin, Brown, Heathcote et al., 2011; Donkin, Heathcote, & Brown, 2009; Ratcliff, 2001; Ratcliff & Smith, 2004; Teodorescu & Usher, 2013; for a diagramatic overview of the relationship between the models, see Ratcliff et al., 2016). The four models were chosen to encompass the range of tasks analyzed in the first part of this article, and because they represent different ways of implementing the mechanisms we are interested in.

**Figure 3 fig3:**
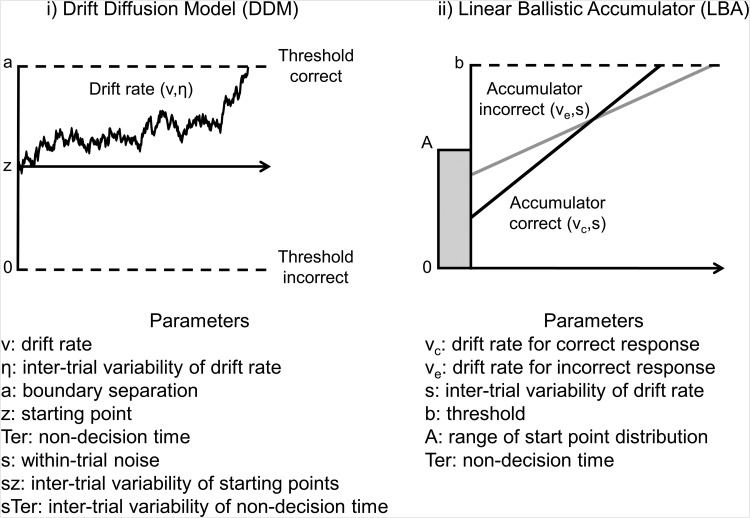
Schematic of two sequential sampling models. i) The drift-diffusion model (Ratcliff, 1978; Ratcliff & McKoon, 2008) consists of a single accumulator accruing evidence from a starting point (z) to one or the other response threshold (a and 0). The drift rate on each simulated trial is taken from a distribution that has a mean (v) and standard deviation (η) across trials, and is subject to within-trial noise (s). ii) The LBA model consists of an accumulator for each response option, accruing evidence to a common response threshold (b). On each simulated trial, drift rates are taken from distributions which have a mean (vc, ve) and standard deviation (s), and begin accumulating evidence from a starting point selected from a uniform distribution (A-0). The models also normally add non-decision time to capture sensory and motor delays, but here we simply assume this is a constant, as variance in non-decision time is not needed for our discussion.

Schematics of the DDM and LBA can be seen in Figure 3. These models assume a constant average rate of evidence accumulation, or drift rate, within each trial. Both also typically assume that drift rates vary between trials, which produces variability in RTs and error rates. A key difference is that drift rates in the DDM are also subject to moment-to-moment noise, which further contributes to variability in performance. In contrast, drift rates are ballistic in the LBA, omitting within-trial noise. A second key difference is that in the DDM, evidence for one response is direct evidence against the alternative, whereas in the LBA the alternative responses have independent accumulators. Though they differ in their structure, both models successfully capture behavioral performance in many cognitive tasks, and broadly lead to the same conclusions about underlying psychological processes (for discussions of issues of complexity and model mimicry, see Donkin, Brown, Heathcote et al., 2011; Donkin et al., 2009; Ratcliff, 2001). The DDM has been employed to explain why individual differences in absolute RT and accuracy did not correlate in numeracy tasks (Ratcliff et al., 2015) and our illustrations below for RT costs and error costs emerge from the same fundamental model properties.[Fig-anchor fig3]

Though the DDM and LBA have been applied to a wide range of tasks, the assumption of constant average drift rate is problematic for many tasks in Table 1, such as the flanker, antisaccade, and Simon, where errors occur mostly on incongruent trials and tend to have short RTs (Gratton, Coles, & Donchin, 1992; Ridderinkhof, 2002). Errors produced by DDM and LBA are normally slow, and although fast errors can be simulated if accumulation start point is given high variability (Heathcote & Love, 2012; Ratcliff & Rouder, 1998), this produces errors on congruent trials as well, because starting point parameters should not vary between intermixed conditions.

**Figure 4 fig4:**
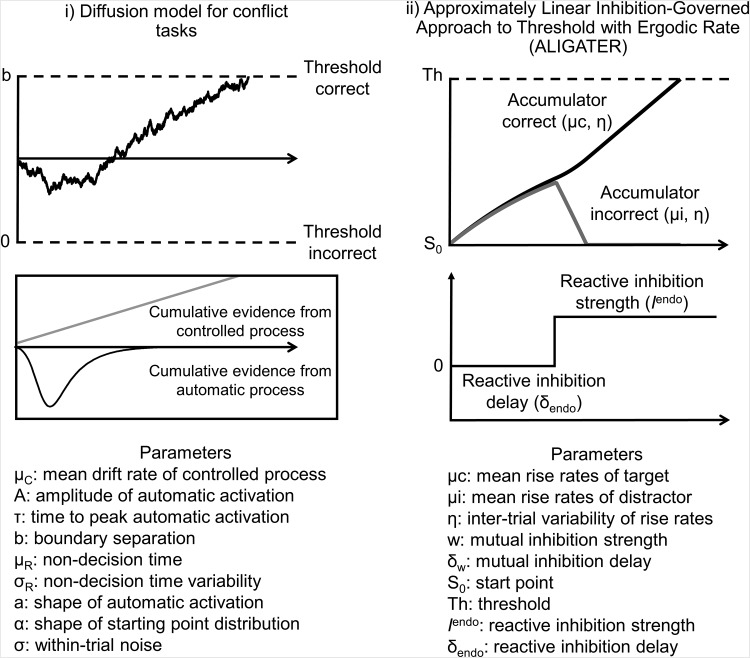
Schematic of two sequential sample models for conflict tasks. i) The diffusion model for conflict tasks, DMC (Ulrich et al., 2015), an extension of the drift-diffusion model to accommodate the flanker and Simon tasks. The DMC adds a transient input for the irrelevant competing information (black gamma function in the lower panel) to the sustained linear process for the correct information (μc: grey line in the lower panel). The gamma function, defined by the parameters A, a and τ, provides an impulse function, so that the irrelevant features (e.g. the flankers) initially have a large input, which diminishes rapidly within the trial. ii) ALIGATER is an extension of LATER (Carpenter and Williams, 1995) originally tested in the context of saccadic interference effects (Bompas & Sumner, 2011). Two LATER units, one for the target and one for the distractor, attempt to rise to threshold while mutually inhibiting each other. To produce goal-directed selectivity ALIGATER includes reactive inhibition instead of altering drift rates. This inhibition attenuates the activation in the distractor node by a specified amount (*I*^endo^) after a delay (δ_endo_) (lower right panel).

Fast errors for incongruent stimuli are taken as evidence for initial automatic activation favoring the prepotent response, which is then inhibited or filtered out on correct trials (Ridderinkhof, 2002; Ridderinkhof, Van den Wildenberg, Wijnen, & Burle, 2004). To capture such dynamics, extensions of the general models have been suggested, such as the DMC and ALIGATER (see Figure 4). The DMC is an extension of the DDM, in which the accumulation rate on each trial combines the normal linear process and a short-lived initial activation for the prepotent response option. ALIGATER is an extension of the LBA and Carpenter and Williams’ (1995) LATER model (linear approach to threshold with ergodic rate). LATER is similar to the LBA, in that it consists of a linear ballistic rise to threshold. ALIGATER extends this by including two types of inhibition: lateral inhibition between accumulators (cf. Usher & McClelland, 2001) and late-starting reactive inhibition to inhibit the incorrect response accumulator. Several other model variants have been proposed and these broadly produce similar patterns of data to the models selected here (see, e.g., Dillon et al., 2015; Hübner et al., 2010; Noorani & Carpenter, 2013; Usher & McClelland, 2001; White et al., 2011).[Fig-anchor fig4]

### Response Selection and Response Caution in the Decision Model Framework

In the decision portion of all of the models outlined above, there are two general factors that influence the nature and the speed of the response. The first is the strength of the evidence or the rate at which the accumulation processes differentiate between correct and incorrect options. This corresponds to the drift rate in DDM, the composite drift rate in DMC, the difference between accumulators’ rates in LBA, and the net effects of accumulation rate, mutual inhibition and reactive inhibition in ALIGATER. This net rate of differentiation can be characterized as *processing efficiency* or *selection*. Differentiation rate clearly changes with the nature of the stimuli: For example, evidence for the “left” response can be more quickly extracted from the flanker congruent stimuli <<<<< than from the incongruent stimuli >><>>.

In most individual differences research, “processing efficiency” maps onto the main construct of interest: the ability to rapidly select the appropriate answer, or the extent to which correct selection is impeded by irrelevant information or prepotent responses. In DDM this would be reflected by different mean drift rates between individuals, in LBA by a larger or smaller difference in accumulation rate for correct and incorrect responses, in DMC by different amplitude in the transient component of drift, and in ALIGATER by reactive inhibition (because this model does not typically include goal-directed bias in underlying accumulation rates for each response option).

The second factor affecting decision speed is how much evidence is required before a decision is made; the threshold or boundary, which has also been described as “*response caution*” (Donkin, Brown, Heathcote et al., 2011). The height of the threshold is thought to be partially under the individual’s control (Ratcliff & Rouder, 1998). In the speed–accuracy trade-off paradigm (Garrett, 1922; Hale, 1969; Wickelgren, 1977; Woodworth, 1899), participants are assumed to set their threshold lower under speed instructions, creating faster responses with a higher risk of errors due to noise or the prepotent signal. Though thresholds can be strategically adjusted, we also assume that individuals vary on their “default” level (Ratcliff et al., 2015). Differences in response caution have been shown to account for group differences that were previously attributed to deficits in processing, for example, in the aging literature (Ratcliff, Thapar, Gomez, & McKoon, 2004; Ratcliff, Thapar, & McKoon, 2006).

Note that models can allow different thresholds for each response, reflecting a bias toward one choice when it is incentivized or more frequent, for example. However, in situations where trials and responses are randomized, unpredictable and equally motivated, no bias is typically assumed, and this is what we assume here.

### Subtracting Performance in a Baseline Condition Does Not Control for Caution

**Figure 5 fig5:**
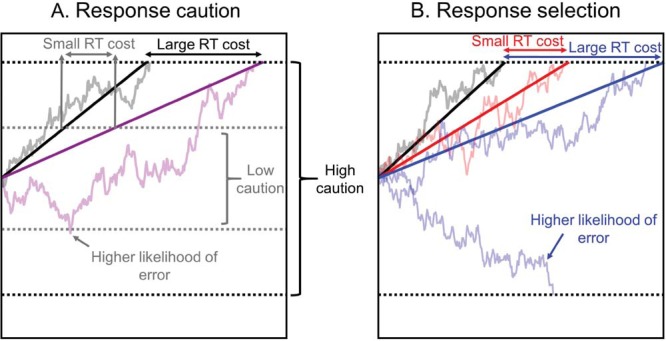
Pattern of RT costs and Error costs produced by variation in response caution and selection in the drift diffusion model. Straight, solid lines show condition averages, faint lines show example individual trials. Black lines show drift rates in congruent/baseline condition, coloured lines show incongruent condition. A. Response caution: Individuals who are low in response caution will set a lower threshold (e.g. grey dotted line) than highly cautious individuals (black dotted line). This means not only that their RTs will be faster, but also the difference between conditions will be smaller, leading to smaller RT costs, noted by grey arrows compared to black arrows. However, the lower threshold will lead to more errors due to noise in the accumulation process, which can be overcome with higher thresholds (example trial in purple reaches the grey error threshold, but not the black error threshold). Note that this will predominantly affect the incongruent or more difficult condition, as errors are rare in congruent/baseline conditions, leading to higher relative error costs. B. Response selection: Individuals who have high selection efficiency will have relatively higher drift rates in incongruent conditions (red solid lines) compared to individuals with lower selection efficiency (blue solid lines), leading to smaller RT costs (noted by red arrows compared to blue arrows). Moreover, the higher drift rate means noise is less likely to cause the incorrect response (illustrated with blue example trial that reaches the error threshold). Note that individuals could also vary in their average drift rates in congruent conditions, and the conclusions would remain the same, since the same difference in drift rate between conditions creates larger costs if average drift rates are lower. For simplicity we keep average congruent drift rates constant in our simulations.

The potential contribution of caution to differences in absolute RT and accuracy (Ratcliff et al., 2015; Thompson, Ratcliff, & McKoon, 2016) is one of the key reasons why many tasks employ a within-subject subtraction between conditions (i.e., the RT cost or error cost). It is commonly assumed that such subtraction controls for speed–accuracy trade-offs, but in accumulation models it does not (see also Ratcliff, Spieler, & McKoon, 2000; White, Curl, & Sloane, 2016). This is in essence the most important difference between the accumulation model framework and traditional conceptualizations of these tasks. In the models, individual differences in threshold will contaminate (or be part of the interesting variance in) RT costs and error costs when attempting to measure individual differences in selection or any other aspect of task performance. Higher levels of selection efficiency lead to both smaller RT costs and smaller error costs. In contrast, higher levels of response caution lead to *larger* RT costs and *smaller* error costs. The mechanisms of this are illustrated for the drift diffusion model in Figure 5 (see Supplementary Material D for other models).[Fig-anchor fig5]

### Simulated Examples

To illustrate the effects of individual variation in response caution and selection efficiency, we simulated the patterns of RT costs and error costs produced by the DDM, the LBA, the DMC, and ALIGATER. Each simulation consisted of 50,000 trials per condition. The ranges of parameters used in our simulations were informed by previous simulations using these models where available, as well as our own simulations. For brevity, we use the terms “congruent” and “incongruent” to refer to all tasks, thus encompassing congruent/baseline/target/valid and incongruent/alternate/distractor/invalid conditions respectively. The general results of our simulations are not dependent on the choice of either a congruent or neutral condition as a baseline (cf. Jonides & Mack, 1984), as the difference between conditions in both cases would typically be captured by differences in processing efficiency.

#### Drift-diffusion model (DDM)

In this model, basic congruency effects are captured by differences between mean drift rates for congruent and incongruent trials (v1, v2). To simulate individual differences in caution, we let boundary separation (a) vary between 0.07 and 0.16 in increments of 0.015. To simulate individual differences in selection efficiency, mean drift rates for incongruent trials varied from 0.1 to 0.4 in increments of 0.05 (while mean drift rates for congruent trials were constant at 0.45). Parameters describing between-trial variability in drift rates (η), mean start point bias (z), and within trial-noise (s) were held constant across simulations (see Table 2 for values used). The DDM was simulated using the DMAT toolbox (Vandekerckhove & Tuerlinckx, 2008) in Matlab, 2014 (The MathWorks Inc. Natick, MA, USA). Parameter ranges were informed by Donkin, Brown, Heathcote, and Wagenmakers (2011).

#### Linear ballistic accumulator model (LBA)

In this model, congruency effects are captured by differences between mean drift rates on congruent and incongruent trials (v1, v2). To simulate individual differences in caution, we varied the response boundary parameter (b) from 250 to 550 in increments of 50. To simulate individual differences in response selection, mean drift rates for incongruent trials varied between 0.95 and 0.65 in increments of 0.05 (mean drift rates for the correct response accumulator on congruent trials were fixed to 1). The drift rates for the incorrect response accumulators were fixed to 1 minus the drift rate for the correct response. Parameters describing start point variability (A) and between trial variability in drift rates (s) were held constant for all simulations (see Table 2). The LBA model was simulated using code provided in R (Donkin, Averell, Brown, & Heathcote, 2009; Donkin, Brown, & Heathcote, 2011), using parameter ranges derived from Donkin, Brown, and Heathcote (2011).

#### Diffusion model for conflict tasks (DMC)

In this model, congruency effects are captured by the amplitude of automatic activation (A for congruent trials, 0–A for incongruent trials). To simulate differences in caution, we varied boundary separation (b) between 35 and 65 in increments of 5. To simulate differences in selection efficiency, we varied the amplitude of automatic activation between 10 and 28 in increments of three. Parameters describing the drift rate for the controlled process (μc), time to peak automatic activation (τ), the shape parameter of the starting point distribution (α), the shape parameter of the automatic activation function (a), and within-trial noise (σ) were fixed for all simulations (see Table 2). The DMC (Ulrich et al., 2015) was implemented in Matlab, using parameter ranges reported by Ulrich et al. (2015) as well as informed by our own simulations.[Table-anchor tbl2]

#### Approximately linear inhibition-governed approach to threshold with ergodic rate (ALIGATER)

In ALIGATER, congruency effects are captured by mutual inhibition and reactive inhibition that selectively inhibits the accumulator for the incorrect response on incongruent trials. Congruent trials consist of a single accumulator with a linear rise to threshold, making the model in these trials equivalent to LATER (Carpenter & Williams, 1995) or LBA without start-point variability. Drift rate for the single accumulator in congruent trials, and for the correct and error accumulators in incongruent trials, are fixed to the same value. To simulate differences in caution, we varied the threshold (Th) between 0.7 and 1.3 in increments of 0.1. To simulate differences in selection efficiency, we varied the strength of reactive (endogenous) inhibition (*I*^endo^) from 0.01 to 0.022 in increments of .002. Parameters describing the mean drift rates (μc, μi), between trial variability in rise rates (η), reactive inhibition delay (δ_endo_), mutual inhibition strength (w), and mutual inhibition delay (δ_w_) were fixed across all simulations (see Table 2). ALIGATER (Bompas & Sumner, 2011) was implemented in Matlab, with parameter ranges informed by Bompas and Sumner (2011), as well as our own simulations.

### Simulation Results

**Figure 6 fig6:**
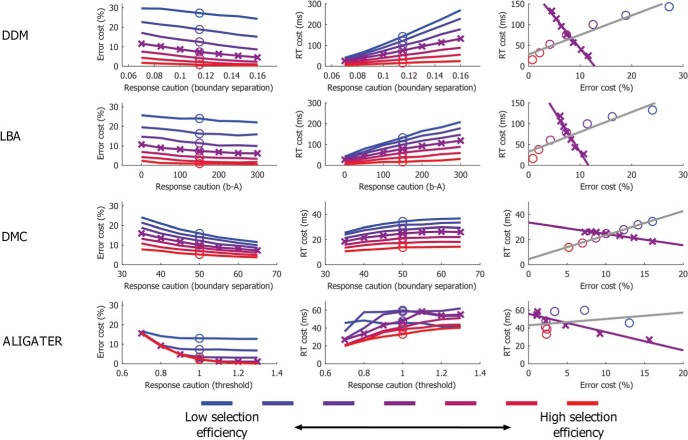
Simulated error costs and RT costs produced by four decision models. DDM = Drift-diffusion model, LBA = Linear ballistic accumulator model, DMC = Diffusion model for conflict tasks, ALIGATER = Approximately linear rise to threshold with ergodic rate. The first and second columns show the patterns of error costs and RT costs, respectively, as a function of variation in both caution and response selection as implemented in the different models (see main text for details). The third column shows the correlation between RT costs and error costs that arise from holding response selection constant and allowing caution to vary (purple line and crosses), and for allowing response selection to vary while caution is held constant (grey line and circles). Though the simulated data are often non-linear, linear trend lines are plotted for illustrative purposes since most studies of individual differences would calculate linear correlations. Note some changes of scale between plots, due to the range of parameters used, as guided by previous literature (see text). Trials with decision times longer than 2000 ms were excluded from the plots.

The relationships between RT cost and error cost from the simulated data are shown in Figure 6. The first column shows the effect of variations in selection efficiency and caution (as conceptualized by each model) on error costs from each model. The second column shows the corresponding effects on RT costs. The third column shows the expected correlation between RT costs and error costs as either caution or selection varies between individuals. For example, the gray line and circle markers in the top right panel shows the effect of varying incongruent drift rates (selection efficiency) in the drift diffusion model while holding boundary separation constant is a positive correlation. These are the data points highlighted by circle markers in columns 1 and 2 (note that individual points also keep their colors when replotted in column 3). The purple line in the top right panel shows the effect of varying boundary (threshold) separation while holding drift rates constant is a negative correlation (drawn from the purple data points marked by crosses in columns 1 and 2).[Fig-anchor fig6]

The critical point to be taken from Figure 6 is that all of the models can account for positive, negative, or absent correlations between RT costs and error costs, depending on whether variance in selection efficiency or in caution dominates (and the ranges of that variance), or whether both vary such that no overall correlation appears. In practice, variance in both caution and selection efficiency is expected in all studies, and the extent to which one or the other dominates may be influenced by population, sampling variance, task, or task instructions (see Part 3). As such, the data in Table 1 is to be expected in this framework. This conclusion is independent of the specific model used.

Though all the models produce similar behavior with respect to the patterns of RT and error costs there are notable differences between models worth explaining. First, as noted when introducing the models, errors are typically fast for ALIGATER and the DMC, while errors tend to be relatively slow in the DDM and LBA. Second, the data are nonlinear to different degrees. For example, the strong nonlinearity in ALIGATER occurs partly because the cost of successfully saving a would-be error is to produce a relatively long correct RT. On a trial with an initially strong level of distractor activation, an individual with low response selection efficiency will make an error. In contrast, an individual with higher levels of response selection efficiency may save the error, but this correct response will be slow due to mutual inhibition from the distractor. Thus, despite high selection efficiency, slow RTs get added to this individuals RT distribution that are absent for the individual with low selection efficiency. Analogous behavior can occur in other models. For example, individuals with higher drift rates in the DDM and LBA are less likely to make errors on trials where start point variation favors the error response, though these trials will produce relatively long RTs (cf. Ratcliff & Rouder, 1998). However, as the average drift rates typically differ between conditions in the LBA and DDM, this behavior has less of an influence on the overall RT distribution.

### Alternative Sources of Slowing and Errors Within the Models

Our simulations focus on the dimensions of response selection and response caution, as they are implemented across many evidence accumulation models. As shown above, these two concepts are sufficient to explain the results of the meta-analysis. However, other parameters in the models also influence RTs and error rates. We conduct additional simulations in Supplementary Material G to illustrate these relationships, and we give an overview of commonly discussed parameters below. For the interested reader, we also examine the influence of varying the time-to-peak parameter in the diffusion model for conflict tasks in Supplementary Material G.

#### Average drift rates or general processing efficiency

We characterize response selection as the difference between evidence accumulation rates in two conditions. This represents an individual’s ability in a particular cognitive domain, for example, in the Stroop task. For two individuals with equivalent drift rates for congruent stimuli, an individual with low selection ability will show lower drift rates for incongruent stimuli relative to an individual with high selection ability. In reality individuals are also likely to vary in their general ability to process information, such that drift rates to congruent and incongruent stimuli would be correlated. The impact of this is that an individual with a lower average drift rate will show larger RT costs and error costs relative to an individual with a higher average drift rate even if they have the same response selection ability (i.e., relative difference between drift rates). This would create correlation between measures. In other words, general slowing can “look like” domain specific deficits in traditional measures.

This also means that traditional analyses of RT costs are difficult to interpret when comparing populations with different mean RTs (see also Faust et al., 1999). But if we assume accumulation models are a meaningful framework, and one has sufficient data to estimate the parameters, individual differences in average processing rates are not distinctly problematic. Drift rates are typically freely estimated for each condition, such that one can formulate hypotheses about the difference between drift rates without confounding or constraining average drift rates.

#### Nondecision time

Nondecision time reflects the total duration of perceptual and motor processes, which often represent a sizable proportion of RTs. Individual differences in nondecision time are therefore highly relevant to attempts to link individual differences in mean RT to constructs such as general intelligence (e.g., Der & Deary, 2017) or mental health (e.g., Gale, Harris, & Deary, 2016; see also Miller & Ulrich, 2013). In most of the paradigms we discuss it is common to assume that nondecision time does not vary between conditions. This reflects an assumption that, for example, early visual processes do not take longer for an incongruent flanker stimulus (<<><<) relative to a congruent stimulus (<<<<<). This simplifying assumption is also made in other models of RT (e.g., Miller & Ulrich, 2013).

As increasing nondecision time is assumed to slow RTs in both conditions equally it would not affect the RT cost. It is also assumed the nondecisional processes do not affect accuracy, so it would not affect the correlation between RT costs and error costs. However, the assumption very much a simplification, and depends on the definition of what is visual processing and what is goal-directed information accumulation. Indeed, this distinction has no clear mapping onto visual information flow through the brain, which is sensitive to attention/relevance from the earliest stages. There are some paradigms where differences in nondecision time between conditions have been explicitly implicated (e.g., masked vs. unmasked priming; Gomez, Perea, & Ratcliff, 2013). In these cases, variation in nondecision time in the slower condition would affect the size of the RT cost without affecting the error cost, diminishing the correlation between RT costs and error costs.

#### Variation in starting points

Another common simplifying assumption in the DDM is to constrain the starting point of the accumulation process to be equidistant between the two response boundaries on every trial. This assumption is typically not made in the LBA, where starting point variability contributes to variation in RTs in the absence of within-trial (diffusion) noise. Starting point variability is often invoked to account for fast errors, which would be likely if the accumulation process sometimes begins close to the boundary for the incorrect response (Heathcote & Love, 2012; Ratcliff & Rouder, 1998; Ratcliff & Tuerlinckx, 2002). This entails that on some trials the accumulation process begins close to the boundary for the correct response, such that a fast correct response is given. As such, it impacts on the RT and error rate of both conditions. Our simulations in Supplementary Material G indicate that it has relatively little impact on the RT cost and error cost.

## Part 3: Testing Predictions of the Accumulation Model Framework

### Prediction 1: Speed Instructions Increase Correlation Between RT Costs and Error Costs

In our simulations, variation in response caution led to negative correlations between RT and error costs, whereas variation in selection efficiency led to more positive correlations. Therefore, the model framework predicts that reducing variability in response caution—and thus increasing the proportion of variance accounted for by selection efficiency—would lead to more positive correlations.

In their examination of the relationship between average accuracy and average RT in numerical cognition, Ratcliff, Thompson, and McKoon (2015) reasoned that, if levels of response caution are flexible, then emphasizing speed in their instructions should reduce variance in response caution relative to encouraging participants to be both fast and accurate (which is often the standard task instruction). If we apply the same logic to the examination of RT and error costs, then we should observe that the correlation between costs is more positive under speed instructions than under standard task instructions. To test this prediction, we draw upon data from two studies recently conducted in our lab for the purpose of examining the reliability and generality of adjustments to caution. In the first study, participants completed the flanker and Stroop tasks in two sessions. In the second, participants completed the flanker and a random-dot motion discrimination task in a single session. Both studies consisted of speed, accuracy, and both speed and accuracy (standard) instruction conditions. Here, we examine whether the correlation between RT and error costs is higher under speed instructions relative to standard instructions. We also report the correlations under accuracy instructions for completeness, but this was not directly compared with the other conditions (see below).

Detailed methods for these experiments are in Supplementary Material E. For brevity, we give an overview here. In the first study, 57 participants performed both the flanker and a manual Stroop task in two sessions taking place 4 weeks apart. In the second study, 81 participants performed the flanker task and a random dot motion discrimination task in a single session. At the beginning of speed-emphasis blocks, participants were asked to “Please try to respond as quickly as possible, without guessing the response.” For accuracy blocks, participants were told “Please ensure that your responses are accurate, without losing too much speed.” For standard instruction blocks, participants were instructed “Please try to be both fast and accurate in your responses.” Feedback was also manipulated to encourage speed and/or accuracy in accordance with the instructions.

### Data Analysis

The same inclusion criteria and RT cut-offs described in Part 1 were applied; the number of participants included in the analysis for each task, session, and study is shown in Table 3.[Table-anchor tbl3]

To test whether the correlation between RT and error costs is more positive under speed instructions relative to standard instructions, we adopted a meta-analytic approach. First, for each dataset, we calculated the Pearson correlation between the RT costs and error costs in speed and standard instruction conditions separately. We then applied the Fisher’s z-transform (Fisher, 1914) to the coefficients, and transformed these back into R values. Treating the differences in R values between instructions as the effects of interest, we then calculated a weighted average effect using Hedges and colleagues’ method assuming a random-effects model (Field & Gillett, 2010; Hedges & Olkin, 1985; Hedges & Vevea, 1998). Note that more complex methods could take into account the nested structure of our data, but we opt for the simpler approach given the small number of data sets.

### Results and Discussion

We limit our coverage of the results to the correlations between RT and error costs. Table 3 summarizes the correlations in each condition, and the difference between the correlations in the standard and speed-instruction conditions. We report the correlation under accuracy instructions for completeness, though following Ratcliff et al. (2015), we restrict our analysis to the comparison of speed emphasis to standard instructions. The weighted average effect size was R = .19 (95% CI [.09, .29], z = 3.57, *p* < .001), indicating that the correlation between RT and error cost is indeed more positive under speed instructions. Note that this effect was fairly consistent, with none of the data sets showing a more positive correlation under standard instructions. This is consistent with the accumulation model framework.

The size of the effect that we observe (.19) is small by commonly used criteria (Cohen, 1988), though we consider it to be meaningful given that the unweighted average correlation under standard instructions in Table 3 was R = .17 (note that this is similar to the average of R = .17 observed in Table 1). Nevertheless, at R = .36, the average correlation under speed instructions was still far from unity. While speed instructions may lessen the impact of variation in response caution, RT and error costs cannot be considered interchangeable.

The enhanced correlations under speed instructions do not arise simply from expanding the variance of the constituent variables; the standard deviation of the RT cost decreased from 30 ms to 18 ms on average, while it increased from 5% to 7% for the error cost (see Supplementary Material E). Note that while our hypothesis was derived from comparing speed with standard instructions as in Ratcliff et al. (2015), performance under standard instructions is often similar to that under accuracy instructions, such that theorists have suggested that the typical default strategy is to minimize errors (Forstmann et al., 2008; van Maanen et al., 2011; van Veen, Krug, & Carter, 2008). There is inconsistent evidence for this in Table 3, but it is not our focus here.

In our logic we assumed that speed instructions both lower thresholds (response caution) and reduce its variance across participants (see Ratcliff et al., 2015). That is not to say that threshold is the only parameter affected by speed–accuracy trade-offs. Several studies suggest that speed instructions may additionally lower drift rates and reduce nondecision time (e.g., Rae, Heathcote, Donkin, Averell, & Brown, 2014; though see Arnold, Bröder, & Bayen, 2015; Starns & Ratcliff, 2014). A reduction in nondecision time should not affect the correlation between RT costs and error costs assuming that it affects both congruent and incongruent conditions equally. A reduction in drift rates with an accompanying increase in drift rate variance would additionally shift the proportion of variance from threshold to drift rate and might be a factor behind the increased correlation.

One might also ask whether these findings are consistent with alternative models of the speed–accuracy trade-off. Two prominent explanations are the fast-guess model (Ollman, 1966, 1970) and the deadline model (Yellott, 1971). The fast-guess model assumes that on some proportion of trials participants do not process the stimulus and instead make a fast guess with a short RT and chance accuracy. This proportion increases under speed instructions. In contrast, the deadline model contains late guesses, whereby participants respond with chance accuracy if a stimulus has not been categorized before some internal cut-off. The deadline model assumes that participants reduce this time limit under speed instructions.

Both models have fallen out of favor in recent years due to their inability to capture data from a range of speed-accuracy experiments (see Heitz, 2014 for a review). Nevertheless, we include simulations of predictions from both models in Supplementary Material I. Briefly, increased correlation between RT costs and error costs under speed emphasis is compatible with a deadline model. Reduced deadline variance acts like reduced threshold variance, limiting the ability of participants to trade errors for longer RTs in the more difficult condition. A fast guess account does not predict a more positive correlation, as an equal number of fast guesses are added to both conditions irrespective of their difficulty.

### Prediction 2: Intermixed Conditions Increase Correlation Between RT Costs and Error Costs

A second prediction of the framework is that tasks with intermixed trial conditions should produce more positive correlations than blocked conditions in the same task. Again, this prediction arises from reducing the contribution of threshold variance to performance variance. Accumulation models generally assume that boundary cannot be changed midway through a trial—and thus unpredictably intermixing trials forces participants to have the same boundary for every trial. On the other hand, explicitly blocking different conditions allows participants more freedom in adopting different levels of caution. The introduction of more freedom translates into more variance between participants.

Anecdotal support for this hypothesis can be found in Table 1, with negative correlations observed in the Navon global precedence and antisaccade tasks, which used blocked conditions. Blocked designs are common in these tasks, where intermixed trials would introduce a rule switching component (blocked designs also occur in IAT tasks, but here participants would not be aware of the blocked arrangement, so the prediction does not apply). To test this prediction, we ran a new study (*N* = 102) using the Simon task. In the same subjects, we compared the correlation between RT costs and error costs when trials are randomly intermixed (as is typical with the Simon task), compared with blocks of congruent and incongruent trials administered separately (e.g., as is common with the antisaccade task). We predicted that the correlation between RT and error costs would be more positive in intermixed trials. Detailed methods are reported in Supplementary Material F. The data are available at https://osf.io/btsrw/.

### Results and Discussion

The correlations between RT and error cost measures can be seen in Table 4, along with the descriptive statistics. Spearman’s correlations are reported due to the presence of an outlier in the blocked condition. The correlations between RT and error costs within blocked and mixed version of the task are highlighted.[Table-anchor tbl4]

As predicted, a modified Pearson-Filon test (Raghunathan, Rosenthal, & Rubin, 1996) showed that the correlation between RT and error costs for mixed trials (ρ = .61) was significantly more positive than that for blocked trials (ρ = −.20), Z = 6.49, *p* < .001. Note that both were significantly different from zero, with a significant negative correlation observed in blocked trials.

Note that the overall error rates and RTs were larger for mixed trials (congruent: 7.9%, 408 ms; incongruent: 11.2%, 429 ms) compared with blocked trials (congruent: 3.2%, 308 ms; incongruent: 6.8%, 354 ms), while RT costs and variance in the RT costs was greater for blocked trials. Therefore, the higher correlation in mixed trials does not arise simply from an increase in variance. This pattern is consistent with participants decreasing their caution (to a variable extent) when they anticipate there will be no difficult trials. The degree to which they do this then drives the correlation between error costs and RT costs. In contrast, where trials are intermixed within blocks, caution cannot be adjusted between trial types, and the correlation between RT costs and error costs are driven more by variation in response selection.

A second notable observation from Table 4 is that neither RT costs nor error costs from the blocked trials correlate significantly with their counterparts from mixed trial blocks. This is highly problematic from the theoretical standpoint that performance in the Simon task simply reflects ability to inhibit a prepotent response. However, it is to be expected if variation in the costs derived from the blocked format are driven more by individual differences in response caution, whereas differences in response selection are more influential in mixed blocks. Note that this could also explain absent correlations between tasks thought to measure the same cognitive ability, but that differ in their blocking structure (e.g., such as between antisaccade and flanker paradigms).

## General Discussion

As psychologists explore what Cronbach (1957) called the “outer darkness” of error variance, it is becoming clear that the relationship between individual differences and experimental research is not always straightforward. Between-subjects variance can arise from different mechanisms to within-subject variance (Borsboom, Kievit, Cervone, & Hood, 2009; Boy & Sumner, 2014), and the average behavior of a group can misrepresent underlying patterns of individuals’ responses (Liew, Howe, & Little, 2016). Here, we demonstrate another counterintuitive finding across psychological paradigms. It is often assumed that subtracting between conditions controls for factors such as speed–accuracy trade-offs. In turn this leads to the widespread assumption that variance between individuals in performance indexes cognitive ability (processing efficiency) in that domain. This is the underpinning of nearly all theory built on individual differences in such tasks—such as the relationships between cognitive domains or with psychiatric disorders. If this were true, alternate measures of performance from the same task should always correlate. Our meta-analysis shows this assumption does not hold across a wide range or tasks.

In the second part of this article, we illustrated how subtractions do not control for threshold (caution) differences within the framework of decision models. In turn, this means such models predict that RT costs or error costs are rarely interchangeable as performance measures—they would only be strongly correlated when threshold variance is very low. Evidence accumulation models provide a theoretical framework across cognitive psychology and cognitive neuroscience (cf., Forstmann & Wagenmakers, 2015; Forstmann et al., 2011; Ratcliff et al., 2016). They have been applied to a wide range of cognitive domains, including memory (Ratcliff, 1978), perceptual decision making (Brown & Heathcote, 2008; Ratcliff & Rouder, 1998; Usher & McClelland, 2001), choice preference (Tsetsos, Usher, & Chater, 2010), language (Brown & Heathcote, 2008; Ratcliff, Gomez, & McKoon, 2004; Wagenmakers, Ratcliff, Gomez, & McKoon, 2008), numeracy (Ratcliff et al., 2015; Thompson et al., 2016), and response control (Gomez, Ratcliff, & Perea, 2007; Ulrich et al., 2015; White et al., 2011). A strength of these models is that they can account for the patterns of behavioral speed and accuracy in conjunction (for a review, see Ratcliff et al., 2016). Increasingly, the models are now being used to understand group differences in clinical contexts (Metin et al., 2013; White, Ratcliff, Vasey, & McKoon, 2010; Zhang et al., 2016). Such an approach seems fruitful for correlational research (e.g., Ratcliff et al., 2015), given evidence presented here and elsewhere that thresholds (or speed–accuracy trade-offs) cannot be equated between individuals through instruction alone (Lohman, 1989; Ratcliff et al., 2015; Wickelgren, 1977).

### Linking Measures to Mechanisms

The decomposition of speeded decisions into (at least) two components does come at a cost of increasing the complexity of interpretations. However, this complexity may be a necessity rather than a handicap. Theorists have noted that there is a tendency in the literature to attribute variation on a given task almost directly to variation in a single cognitive function, such as executive control, numeracy, or inhibition (Monsell & Driver, 2000; Ratcliff et al., 2015; Verbruggen, McLaren, & Chambers, 2014). Verbruggen, McLaren, and Chambers (2014) argue that this often results in a redescription of tasks or manipulations, rather than an explanation of the mechanisms underlying performance. Similarly, Ratcliff et al. (2015) argued that the absence of a theoretical model of decision making in numeracy judgments made accounting for inconsistent relationships between RT and accuracy measures problematic. Ratcliff et al. (2015) further proposed that the DDM provided such a theory, within which performance on numerical tasks can be understood. Evidence accumulation models explicitly remind us that manipulations are rarely process-pure (Forstmann et al., 2016; Forstmann & Wagenmakers, 2015). As with any formal model, one can quantitatively test whether an experimental manipulation taps selectively into an underlying parameter of interest. Where a manipulation is not process pure, one can dissociate the effects on the underlying processes, for example, by examining differences in fitted drift rates rather than raw RT or error measures.

We expand upon these recommendations in three key ways. First, we focus on the common practice of subtracting one condition from another, which is often assumed to control for differences in caution. Second, we demonstrate that inconsistent relationships between effects in RTs and effects in accuracy are widespread. These inconsistencies permeate domains of psychology that are at the forefront of initiatives focused on understanding cognitive deficits in clinical conditions, such as executive control, attention and response inhibition (e.g., Barch, Braver, Carter, Poldrack, & Robbins, 2009; Nuechterlein, Luck, Lustig, & Sarter, 2009).

Third, we demonstrate that interpreting correlations between RT costs and error costs with respect to mechanisms of response selection and response caution is not specific to a given model. It has been noted that there is a high level of mimicry between the LBA and DDM, and that despite different architectures, often one would interpret effects with respect to the same underlying processes (Donkin, Brown, Heathcote et al., 2011). The DMC (Ulrich et al., 2015) and ALIGATER (Bompas & Sumner, 2011) models are nonlinear departures from these general frameworks. The DMC and ALIGATER contain mechanisms such as transient excitation or inhibitory control, and produce different patterns of behavior compared with the DDM and LBA. Nevertheless, in terms of the fundamental issue at stake here, parameters reflecting response caution and selection efficiency influence performance similarly across all these models.

Decision models also allow for other mechanisms to be incorporated. For example, biases due to stimulus probabilities or incentives (e.g., Leite & Ratcliff, 2011) can be captured by relative starting point bias in the DDM, or equivalents in other models. However, while models may account well for phenomena at a behavioral level, they may not map directly on to functioning at a neurophysiological level (Heitz & Schall, 2012). Neurophysiological measures can provide useful tests of model assumptions (see, e.g., Bompas, Sumner, Muthumumaraswamy, Singh, & Gilchrist, 2015; Burle, Spieser, Servant, & Hasbroucq, 2014; Servant, Montagnini, & Burle, 2014), and therefore may be useful in guiding and constraining cognitive models (Forstmann & Wagenmakers, 2015).

### Response Caution and the Speed–Accuracy Trade-Off

Considering speed and accuracy in conjunction has a long history in psychology in the context of the speed–accuracy trade-off (SAT; Garrett, 1922; Hick, 1952; Pachella, 1974; Wickelgren, 1977; Woodworth, 1899). Pachella (1974) noted that the assumption behind many RT measures, that RTs reflect the minimum duration required by participants to perform the task at maximum accuracy, is often untested and likely untrue. Wickelgren (1977) argued “. . . the case for speed-accuracy tradeoff as against reaction time is so strong that this case needs to be presented as forcefully as possible to all cognitive psychologists” (p. 68). He went on to acknowledge that the requirement for additional trials over standard designs limited the appeal of trade-off designs, and noted that when considering mean differences between conditions: “When both errors and reaction times go in the ‘same’ direction, then it is reasonably safe to conclude that the condition which is slower and has more errors is more difficult than the condition that is faster and has fewer errors” (p. 79). Our analysis demonstrates that establishing the same directionality of effects at the group level does not entail that both RT costs and error costs will rank individuals equivalently. Indeed, as we show in Part 3, a commonly used design practice (blocking conditions) can create a negative correlation between them. As such, researchers should not assume that RT costs and error costs derived from blocked methods predominantly reflect response selection mechanisms. We recommend that the correlation between RT and error costs be reported, and that explicit consideration be given where effects are examined/observed in one measure and not the other.

For many research questions, response caution might be considered a nuisance parameter that confounds the effect of interest. For example, if a researcher is interested in individual differences in attention, then they are likely interested in the efficiency of information processing, either on average or with respect to some stimulus manipulation. This is the very logic behind subtracting between conditions, which was assumed to allow such processes to be examined in isolation. But caution is an interesting and fundamental component of decision-making. A wealth of literature exists examining the cognitive and neurological mechanisms underlying response caution, in both clinical and nonclinical populations (Dutilh, Forstmann, Vandekerckhove, & Wagenmakers, 2013; Dutilh et al., 2012; Metin et al., 2013; Moustafa et al., 2015; Starns & Ratcliff, 2010, 2012; van Maanen et al., 2011; Zhang & Rowe, 2014). For some research areas, such as the study of impulsive behaviors, the extent to which individuals are willing to commit errors for the sake of faster RTs is of distinct theoretical interest.

For decision models themselves, there is an ongoing debate whether caution is adequately captured by a simple threshold that does not vary within trials. For example, mechanisms by which the level of required evidence decreases over time have been proposed (Bowman, Kording, & Gottfried, 2012; Cisek, Puskas, & El-Murr, 2009; Ditterich, 2006; Drugowitsch, Moreno-Bote, Churchland, Shadlen, & Pouget, 2012; Thura, Beauregard-Racine, Fradet, & Cisek, 2012). These proposals take the form of either a collapsing boundary, or an urgency signal that increases the rate of evidence accumulation. A recent review found that most human data was best accounted for with fixed thresholds, though evidence for dynamic thresholds was observed in nonhuman primates (Hawkins, Forstmann, Wagenmakers, Ratcliff, & Brown, 2015). In many (but not all) of the tasks we discuss, trials are typically randomly presented within blocks, and thus it is assumed that caution does not change between congruent and incongruent trials. Therefore, at a within-subject level, both RT costs and error costs in response control tasks arise from differences in drift rates (or parameters that affect relative accumulation rate) between conditions. However, at a between subject level, the magnitude of an individual’s RT cost and error cost is a reflection of both their level of response caution and of response selection.

### Model Similarities and Differences

Our simulations cover only a selection of evidence accumulation models used in the literature, though most models implement mechanisms of response selection and response caution in comparable ways. For example, the leaky competing accumulator (LCA; Usher & McClelland, 2001), implements response selection via a relative difference between the inputs (thus drift rates) in a similar approach to the DDM and LBA. The LCA also has a criterion parameter, which is equivalent to the implementation of response caution in the models simulated here. White, Ratcliff, and Starns (2011) recently proposed a modified diffusion model of the flanker task, in which the drift rate varies over time according to a narrowing “attentional spotlight.” The shrinking spotlight, implemented as a Gaussian weighting centered on the central arrow and initially encompassing the flankers, allows the model to capture the fast errors typically observed in the flanker task. Though conceptually different, the resultant dynamics of the model are similar to the DMC, presented here. Therefore, our conclusions extend beyond the models featured in our simulations.

We selected four distinct models to illustrate common behavior, not to emphasize any differences. It is also worth noting that some apparent differences between models are just different ways of achieving a similar goal. For example, ALIGATER contains an explicit selective inhibition mechanism, whereas inhibition is implicit in the DMC. Both amendments to the basic models were introduced to ensure nonlinear dynamics—that initial strong support for irrelevant information diminishes while support for relevant information is maintained. The DMC is thus compatible with an explicit mechanism of top-down inhibition (Ulrich et al., 2015).

Some model differences reflect the task or modality in which the model is typically applied. For example, ALIGATER simulations assume equal mean initial rise rates for both target and distractor; an assumption also made by other models of eye movement tasks (e.g., Noorani & Carpenter, 2013), where accumulation is conceptualized as stimulus-driven. This assumption in turn creates the need for an additional mechanism to select target from distractor. In contrast, the DDM, LBA, and DMC implement a difference in the mean drift rates for correct and incorrect responses. This corresponds to conceptualizing evidence accumulation at the level of relevant information for response selection, rather than direct sensory drive (cf. Sternberg, 2001).

The distinction between these models and their applications is not always clear cut, however (Carpenter & Reddi, 2001; Ratcliff, 2001), and neither do we believe the distinction between perception and response selection is clear cut in the brain. Processes such as attention act at multiple stages of processing, for example (Awh, Belopolsky, & Theeuwes, 2012). Further, not only the stimuli used, but also the requirements of information extraction across different task conditions will differentially draw on different visual pathways—all of which have different delay times (Bompas & Sumner, 2009, 2011).

The assumptions made about perceptual (i.e., nondecision) processes have theoretical implications. For example, the distributional shape of nondecision time variability has recently been questioned (Verdonck & Tuerlinckx, 2016). Whereas nondecision time is typically fixed, or assumed to follow a normal or uniform distribution, Verdonck and Tuerlinckx (2016) suggest that nondecision time may often be right-skewed. This misspecification can impact on the estimates of other parameters (e.g., individual differences in response caution).

Even more counterintuitively for cognitive scientists, using different response modalities (e.g., hands, eyes, or speech) changes the sensory part of nondecision time, with knock-on consequences for response selection phenomena (Bompas, Hedge, & Sumner, 2017). This is because different motor selection areas receive different connections from the various perceptual pathways. In turn, this provides an avenue for linking cognitive process models to neurophysiological models (e.g., Nunez, Vandekerckhove, & Srinivasan, 2017). Though it is clear that there is much to be understood about the properties of decisional and nondecisional time, the pursuit of these questions is aided by theoretical frameworks within which to consider them.

### Alternative Explanations: RT and Error Costs Reflect Different Mechanisms?

Absent correlation between RT costs and error costs in the Stroop task was previously noted by Kane and Engle (2003), who attributed the two effects to different mechanisms. In line with the traditional account of Stroop interference, they argued that RT costs arose from the time taken for conflict resolution, but that errors arose from a failure of goal maintenance. In a series of experiments, they manipulated the proportion of congruent trials in the Stroop task, and additionally measured participants’ working memory (WM) span. When the Stroop task was made up of 75% or 80% congruent trials, low WM span participants made a greater number of errors compared with high WM span participants. When 0% or 20% of trials were congruent, low WM span individuals did not make more errors, but showed increased RT costs. The authors argued that when the proportion of congruent trials was high, low WM span participants would sometimes fail to maintain the relevant task goal (naming the color). The interpretation that errors reflect a failure of goal maintenance has been influential in interpreting differences in clinical groups, for example, where it has been observed that errors and error costs in the Stroop task are predictive of conversion to Alzheimer’s disease in older adults (Balota et al., 2010; Hutchison, Balota, & Duchek, 2010).

These effects could also be described within a decision model framework, given that we would expect individuals to adopt different levels of caution in blocks of different congruency proportions (e.g., Part 3 above). A previous study examining the relationship between diffusion model parameters measured from choice RT tasks and a latent WM factor observed a positive correlation between WM and drift rate, and a negative correlation between WM and boundary separation (Schmiedek, Oberauer, Wilhelm, Süss, & Wittmann, 2007). Thus, individuals with a high WM may have high selection efficiency, and can set a relatively low threshold even when incongruent trials are frequent. In contrast, individuals with low WM span may have low selection efficiency, and would need to be more cautious when incongruent trials are frequent (increasing RT costs). More broadly, an interpretation that errors reflect attention lapses is compatible with decision model frameworks if one applies this interpretation to individual trials in which the drift rate is low (McVay & Kane, 2012).

### Combining RT and Error Measures: Alternatives to Modeling

In the domain of task switching, the reliability and validity of the traditionally used RT costs has also been questioned (Draheim et al., 2016; Hughes et al., 2014). These discussions are based on the explicit assumption that speed–accuracy trade-offs can contaminate RT costs, which are traditionally used in task-switching, and may mask correlations with theoretically related constructs. In two experiments, Hughes et al. (2014) assessed three alternative scoring measures that combine effects in RT and accuracy into a single metric. The alternative scoring methods were: a rate residual scoring method (Was & Woltz, 2007; Woltz & Was, 2006), a binning procedure, and inverse efficiency scores (IES; Townsend & Ashby, 1978, 1983). In Hughes et al.’s (2014) first comparison all three metrics showed similar reliability to the RT cost, with the error cost performing poorly. In Experiment 2, the alternative metrics were superior to the traditional measures. The authors argued that the rate residual and binning methods also showed increased validity because they showed larger associations with other executive functioning tasks than did the traditional measures. Other studies have also observed increased correlations between tasks when using the binning procedure (Draheim et al., 2016) or IES (Khng & Lee, 2014) compared with traditional scoring methods (see Vandierendonck, 2017 for a recent comparison of different composite measures).

These methods are not without their criticisms, however. The use of residual scores as an alternative to difference scores have a long history (Cronbach, 1957; DuBois, 1957), though their practical advantages are not uniform, and their validity and interpretation has been questioned (for a review, see Willet, 1988). Potential inconsistencies and limitations of the binning method (Draheim et al., 2016) and the IES (Bruyer & Brysbaert, 2011) have also been discussed. As noted by Draheim et al. (2016), the binning method requires a somewhat arbitrary decision about the extent to which errors are penalized relative to RTs. It has been argued that the IES should only be used where strong, positive correlations are observed in RTs and errors (Bruyer & Brysbaert, 2011; Townsend & Ashby, 1978, 1983), which our analysis illustrates is not usually the case.

Perhaps the largest advantage the decision model framework has over these alternative scoring methods is that the composite scores lack a theoretical justification for their respective methods of combing accuracy and RT into a single metric (Lohman & Ippel, 1993; Rach, Diederich, & Colonius, 2011). Lohman and Ippel (1993) suggest that there are at least three types of errors—those due to ability, those due to the SAT, and those due to extraneous factors such as lapses of attention. Therefore, it may not be appropriate to treat all errors as equal for the purpose of combining them with RTs. In the decision model framework whether errors are fast or slow has important implications, and thus fitting takes into account not only the error rate but also the RT of each error. Further, increased correlations obtained from composite scores may in fact reflect commonalities in strategy (i.e., response caution) across different tasks, rather than the construct of interest. In summary, we see value in easy to calculate metrics that take both RT and error rates into account, however, we recommend caution in their interpretation in the absence of a specified theoretical framework. Decision models provide such a framework, within which we can account for error rates, as well as the RTs of both correct and incorrect responses.

### Relationship Between Models of Response Selection and Other Models of RT

We have discussed the correlation between RT costs and error costs in the context of evidence accumulation models, though theorists have raised concerns about the interpretation of RT measures outside of this framework (e.g., Faust et al., 1999; Miller & Ulrich, 2013; Sriram, Greenwald, & Nosek, 2010). Further, theorists may not wish to commit to the assumptions underlying any particular formal model of the processes underlying RT and accuracy. However, the principles of evidence accumulation and threshold are compatible with general models of RT. Miller and Ulrich’s (2013) IDRT model proposes that an individual’s average RT and RT costs arise from processing across perceptual input, response selection, and motor output stages. These stages correspond to the nondecision (perceptual + motor time) and decision components in models such as the DDM. Indeed, Miller and Ulrich (2013) note that the response selection stage could be realized as a diffusion or linear accumulation process, but their framework is agnostic to the nature of the processes underlying response selection.

Where Miller and Ulrich’s (2013) work and ours overlap is that they note that an RT cost cannot be simply interpreted as an index of response selection ability, and that it is influenced by other properties such as general processing speed (as we discuss above). A similar point is made by Faust, Balota, Spieler, and Ferraro (1999), who propose a rate and amount model (RAM) of RTs. Here, again, the concepts of rate and amount are comparable to the accumulation of evidence to a threshold, though the RAM does not explicitly model these processes. Faust et al. (1999) propose a method for correcting RT costs for overall RT in the context of aging studies, where the issue of RT costs being positively correlated with average RT has been discussed frequently (Ratcliff et al., 2000; Salthouse, 1996; Verhaeghen, 2014). Again, they make the point that a raw RT cost cannot be simply interpreted as an index of ability in a given cognitive domain.

While both the IDRT and RAM frameworks broadly capture how the latencies of different stages contribute to RT measures, they are agnostic to the nature of the cognitive processes underlying response selection. Further, they do not discuss the relationship between RT and accuracy. This is because both frameworks assume tasks are performed with minimal errors. Miller and Ulrich (2013) note that in order to consider the relationship with accuracy one needs an explicit model of response selection, such as those we discuss here (p. 844). More broadly, our discussion focuses on the assumption that individuals with higher levels of ability in a given domain should be both relatively faster and more accurate (see also Ratcliff et al., 2015). The results of our meta-analysis in Part 1 are at odds with this assumption and theories of response selection provide one way in which these inconsistencies can be understood.

## Conclusions

In reflecting on the divide between individual differences and experimental research, Borsboom, Kievit, Cervone, and Hood (2009) suggest the two approaches are inevitably looking at different levels of explanation. At first glance, this appears to hold true for the data we discuss, where interpretations of behavior at a within-subject level do not easily translate to interpreting between-subjects variation. However, we believe that our findings show one way in which this “outer darkness” can be illuminated. The decision model framework allows the counterintuitive patterns of within- and between-subjects variances to be reconciled.

## Supplementary Material

10.1037/bul0000164.supp

## Figures and Tables

**Table 1 tbl1:** Pearson’s r and Spearman’s rho Correlations Between Reaction Time Costs and Error Costs in Cognitive Tasks

	Study	Task/effect	*N*	Trial *N* (baseline/alternate)	Pearsons’ *r*	Spearman’s rho
Our data and unpublished data	Hedge, Powell, and Sumner (2017)	Flanker (arrows)	104	480/480	.28	.27
		Stroop	103	480/480	.27	.29
		SNARC	40	640/640	.20	.20
		Navon – local conflict	40	320/320	.41	.32
		Navon – global conflict	40	320/320	−.11	−.06
		Navon – global precedence	40	320/320	−.25	−.23
	Other data	Flanker (arrows)	50	336/336	.23	.21
		Simon	50	336/336	.47	.54
		Antisaccade	48	200|400/300|400^†^	−.13	−.18
		Illogical rule task	44	200/200	−.20	−.12
		Distractor	48	200/200	.38	.30
		Antisaccade	21	400/400	−.13	−.15
New analysis of published data	Aichert et al. (2012)	Antisaccade	502	60/60	.20	.22
	Balota et al. (2007)	Lexical decision task (English)	809	1,686/1,686^‡^	.34	.37
	Braem (2017)	Task switching	49	78/78	−.07	.02
	Bugg and Braver (2016)	Exp. 1 Task switching	52	185/199^†^	.10	−.08
		Exp. 1 Rule congruency	52	192/192	.11	.23
		Exp. 1 List congruency	52	192/192	−.13	.18
		Exp. 2 Task switching	32	225/223^†^	.24	.30
		Exp. 2 Rule congruency	32	224/224	.18	.32
		Exp. 2 List congruency	32	224/224	−.37	−.09
		Exp. 3 Task switching	32	123/131^‡^	.22	.26
		Exp. 3 Rule congruency	32	380/126^‡^	.33	.26
		Exp. 3 Incentive	32	46/47^‡^	.07	.02
		Exp. 3 Mixed task	32	252/123	.43	.26
	Chen et al. (2015)	Flanker	42	120/120	.14	.25
	Cherkasoava et al. (2002)	Task switching (antisaccade)	18	104/104	−.14	−.21
	Chetverikov et al. (2017)	Flanker (colour)	58	120/120	.09	.13
	De Simoni and Von Bastian (2018)	Simon	216	192/192	.33	.38
		Stroop	216	192/192	.21	.19
		Numeric Stroop	216	192/192	.24	.19
		Navon (conflict)	216	192/192	.52	.27
		Task switching (animacy/size)	216	128/128	−.03	.01
		Task switching (shape/colour)	216	128/128	.02	−.01
		Task switching (parity/magnitude)	216	128/128	.02	.04
		Task switching (fill/frame)	216	128/128	.07	.05
		Task mixing (animacy/size)	216	512/128	.15	.20
		Task mixing (shape/colour)	216	512/128	.08	.15
		Task mixing (parity/magnitude)	216	512/128	.19	.18
		Task mixing (fill/frame)	216	512/128	.05	.21
	Ebersole et al. (2016)	Stroop	3,305	21/42	.15	.12
	Elchlepp, Best, Lavric, and Monsell (2017)	Task switching	21	878/438^‡^	.11	.08
	Ferrand et al. (2010)	Lexical decision task (French)	868	1,000/1,000	.55	.55
	Gonthier, Braver, and Bugg (2016)	Stroop (picture/word)	95	600/600	.28	.31
	Guye and Von Bastian (2017)	Flanker	142	192/192	−.45	−.08
		Simon	142	192/192	.38	.43
		Stroop	142	192/192	.46	.37
		Task switching (animacy/size)	142	128/128	.15	.12
		Task switching (shape/colour)	142	128/128	.09	.16
		Task switching (parity/magnitude)	142	128/128	.18	.13
		Single vs. mixed task (animacy/size)	142	512/128	−.13	−.09
		Single vs. mixed task (shape/colour)	142	512/128	.26	.16
		Single vs. mixed task (parity/magnitude)	142	512/128	.02	.04
	Hefer, Cohen, Jaudas, and Dreisbach (2017)	Flanker	73	60/60	−.02	.02
		Flanker	64	72/72	.04	.13
	Kelly, Uddin, Biswal, Castellanos, and Milham (2008)	Flanker	26	24/24	.29	.16
	Keuleers, Diependaele, and Brysbaert (2010)	Lexical decision task (Dutch)	39	14,089/14,089	.73	.71
	Keuleers, Lacey, Rastle, and Brysbaert (2012)	Lexical decision task (English)	79	14,365/14,365	.78	.81
	Klein, Liu, Diehl, and Robinson (2017)	*Stroop*	*276*	*80/80*	*.10*	*.02*
	Klemen, Verbruggen, Skelton, and Chambers (2011)	Flanker (gratings)	18	48/48	−.24	−.12
	Kreitz, Furley, Memmert, and Simons (2015)	Flanker	120	50/50	.22	.26
		Flanker	197	50/50	.24	.22
	Mennes et al. (2013)	Simon	21	96/96	.29	.21
	Perrone-Bertolotti et al. (2017)	Manual “antisaccade”	44	256/256	−.03	−.11
		Task mixing	44	256/256	.03	.03
	Rusconi, Dervinis, Verbruggen, and Chambers (2013)	SNARC	17	56/56	.31	.28
	Sandra and Otto (2018)	Stroop	57	90/30	.48	.49
		Task switching	62	130/148^‡^	.21	.21
		Reward	62	140/140	.41	.50
	Saunders, He, and Inzlicht (2015)	Flanker	56	250/250	.28	.36
		Flanker	58	250/250	.58	.59
	Saunders, Milyavskaya, Etz, Randles, and Inzlicht (2018)	Stroop	217	288/288	.28	.29
		Flanker	2,249	50/50	.51	.39
	Von Bastian et al. (2016)	Flanker	120	48/48	−.05	−.08
		Simon	120	150/50	.15	.22
		Numerical Stroop	120	48/48	.36	.31
		Task switching (animacy/size)	120	64/64	−.08	−.09
		Task switching (colour/shape)	120	64/64	.07	−.02
		Task switching (parity/size)	120	64/64	−.18	−.17
		Single vs. mixed task (animacy/size)	120	256/64	.09	.10
		Single vs. mixed task (colour/shape)	120	256/64	.14	.12
		Single vs. mixed task (parity/size)	120	256/64	.03	.11
	Wöstmann et al. (2013)	Flanker	23	80/80	−.13	.08
		Simon	23	320/120	.35	.26
	Xu, Nosek, and Greenwald (2014)	Age implicit association test (IAT)	98,1873	40/40	.27	.38
		Arab IAT	33,8103	40/40	.07	.17
		Asian IAT	37,4882	40/40	.18	.26
		Disability IAT	30,9792	40/40	.26	.37
		Gender-career IAT	85,2861	40/40	.18	.28
		Gender-science IAT	63,6003	40/40	.19	.28
		Native American IAT	21,7444	40/40	.21	.28
		President IAT	37,9465	40/40	.21	.31
		Race IAT	3,339,097	40/40	.30	.40
		Religion IAT	169,247	40/40	.18	.28
		Sexuality IAT	1,452,795	40/40	.24	.35
		Skin color IAT	872,781	40/40	.26	.36
		Weapons IAT	534,563	40/40	.21	.32
		Weight IAT	969,372	40/40	.26	.36
	Zwaan et al. (2017)	Flanker	160	64/64	−.08	−.09
		Simon	160	92/92	.43	.42
Previously reported correlations	Manoach et al. (2002)	Task switching (antisaccade) – schizophrenia	21	104/104	−.05	
		Task switching (antisaccade) – controls	16	104/104	.22	
	Draheim, Hicks, and Engle (2016)	Task switching	552	96/96	−.08	
	Hughes, Linck, Bowles, Koeth, and Bunting (2014)	Task switching	1,902	46/98		.01
		Task switching	46	264/120		.21
	Kane and Engle (2003)	Stroop	87	36/36	−.02	
		Stroop	88	36/36	.17	
		Stroop	138	36/36	.10	
	MacLeod et al. (2010)	Attention networks test: Alerting	1,129	72/72	−.10	
		Attention networks test: Orienting	1,129	72/72	.05	
		Attention networks test: Executive	1,129	96/96	.21	
	Paap and Sawi (2014)	Manual “antisaccade”	117	30/60	.29	
	Rondeel, van Steenbergen, Holland, and van Knippenberg (2015)	Stroop	35	54/54	.20	
	Wylie et al. (2009)	Flanker (Parkinson’s patients)	50	103/103	−.39	
*Note.* Unpublished data refer to data collected in our own laboratory, in part to address this topic. New analysis of published data refer to analyses conducted by us of published datasets that we were able to obtain (see Method section). Previously reported correlations refer to correlations included in published article, for which we conducted no additional analyses. Where authors are italicized, correlations are calculated from summary data, rather than trial by trial data. A version of this dataframe for analysis can be found at https://osf.io/btsrw/.						
^†^ Dataset combines two groups of participants who underwent the same procedure with different trial numbers. ^‡^ Trial numbers varied between participants due to task design (e.g. randomized trials). Averages are reported. The correlations in Draheim et al. (2016) were reported for data from an article under review (Shipstead et al., 2015, as cited in Draheim et al., 2016). The word versus nonword effects are reported for lexical decision tasks.						

**Table 2 tbl2:** Parameters Used for Model Simulations

Model	Response selection	Response caution	Other parameters				
DDM	**Incongruent drift rate (v2)**	**Boundary separation (a)**	Congruent drift rate (v1)	Variability in drift rates (η)	Start point bias (a/z)	Within-trial noise (s)	
	**.1–.4**	**.07–.16**	.45	.1	.5	.1	
LBA	**Incongruent drift rate (V2)**	**Threshold (b)**	Congruent drift rate (v1)	Variability in drift rates (s)	Variability in start points (A)		
	**.95–.65**	**250–550**	1	.27	250		
DMC	**Amplitude of automatic activation (A)**	**Boundary separation (b)**	Controlled process drift rate (μc)	Time-to-peak of automatic activation (τ)	Start point shape (α)	Within-trial noise (σ)	Automatic activation shape (a)
	**10–28**	**35–65**	.63	90	2	4	2
ALIGATER	**Reactive inhibition strength (***I*^endo^**)**	**Threshold (Th)**	Drift rates (μc, μi)	Variability in drift rates (η)	Mutual inhibition strength (w)	Mutual inhibition delay (δ_w_)	Reactive inhibition delay (δ_endo_)
	**.01–.022**	**.7–1.3**	.0078	.0039	.01	1 ms	70 ms
*Note*. Parameters that are varied in simulations are denoted in bold. Ranges and fixed values were informed by previous simulations and implementations of each model in the literature (Bompas & Sumner, 2011; Donkin et al., 2011; Ulrich et al., 2015) and parameters are not intended to be compared across models, but simply supplied for information.							

**Table 3 tbl3:** Sample Sizes and Pearson’s r Correlations Between RT and Error Costs from Studies 1 and 2

Dataset	*N*	Instruction condition			Speed-standard
		Speed	Standard	Accuracy	
Flanker 1 Session 1	55	.56	.36	.31	.24
Flanker 1 Session 2	47	.40	.34	−.01	.07
Stroop 1 Session 1	52	.19	.19	.21	.00
Stroop 1 Session 2	46	.33	.15	.21	.19
Flanker 2	81	.46	.23	.01	.26
Dot-motion 2	73	.22	−.07	−.04	.28
*Note.* Standard-speed instruction coefficients are the difference between the Fisher’s *z*-transformed coefficients. See Supplementary Material I for scatter plots.					

**Table 4 tbl4:** Spearman’s Correlations Between RT Costs and Error Costs in the Simon Task in Study 3 (N = 102)

Measure/condition	RT cost–mixed	Error cost–mixed	RT cost–blocked	Error cost–blocked
RT cost–mixed		**.61**	.10	.10
Error cost–mixed			−.02	.14
RT cost–blocked				**−.20**
Mean	20 ms***	3.4%***	46 ms***	3.6%***
Std. dev	17 ms	5.0%	24 ms	5.0%
*Note*. “Mixed” refers to the costs calculated from blocks in which congruent and incongruent trials are intermixed. “Blocked” refers to costs calculated from separate blocks of congruent and incongruent trials. The bold cells contain the correlations central to our hypothesis.				
* *p* < .05. *** *p* < .001.
