# Supplementary material for: Low and Variable Correlation Between Reaction Time Costs and Accuracy Costs Explained by Accumulation Models: Meta-Analysis and Simulations
Source: Psychol Bull. 2018 Sep 27;144(11):1200–27. doi: 10.1037/bul0000164 (PMC6195302; doi:10.1037/bul0000164)
Supplement: Supplementary file 1 [file Supplemental-Materials_0875.docx]

**Supplementary Material A.**

In the main text, we refer to two previous meta-analyses (Geurts et al., 2014; Mullane et al., 2009) that incorporate both RT costs and error costs. Geurts et al. (2014) performed a qualitative review and quantitative meta-analysis of response inhibition and distractor interference in autistic spectrum disorders (ASD). The studies that they included are listed in their Table 1.

Supplementary Table A1 lists the studies included in their meta-analysis that administered computerised single-trial variants of the flanker, Stroop or Simon tasks. Note, Geurts et al.’s analysis included other tasks which are not suitable for our purposes, such as the go/no-go task and card variants of the Stroop. For each of these studies, we noted whether a significant (or marginally significant) group difference in the RT costs or error costs are reported.

The ASD group showed larger RT costs in 5 datasets, and larger error costs in 3 datasets. No datasets showed effects in both RT costs and error costs. In two datasets, the ASD group showed *smaller* RT costs than healthy controls.

Table A1. Summary of reported RT costs and error costs from Guerts et al. (2014) meta-analysis of response inhibition in ASD

| Paper | Task (stimuli) | Effect in RT cost | Effect in error cost |
| --- | --- | --- | --- |
| Adams & Jarrold ,2012 | Flanker | No | Yes |
| Christ et al., 2007 | Stroop | No | No |
|  | Flanker (shapes) | Yes | No |
| Christ et al., 2011 | Flanker (figures) | Yes | No |
| Dichter & Belger, 2007 | Flanker (Arrows and faces) | No | No |
| Dichter & Belger 2008 | Flanker (Faces) | Yes* | No |
| Geurts et al., 2008 | Flanker (Figures) | Yes | No |
| Henderson et al., 2006 | Flanker | Yes* | No |
| Larson et al., 2012 | Flanker | No | Yes |
| Robinson et al. 2009 | Stroop | No | Yes |
| Russel et al 1999 | Stroop (figures) | No | No |
| Schmitz et al., 2006 | Simon/Stroop | No | No |
| South et al., 2010 | Flanker | No | No |
| Vaidya et al., 2011 | Simon/flanker | Yes | No |
| Xiao et al., 2012 | Stroop | Not Reported | Not Reported |
| Yoran-Hegesh et al., 2009 | Stroop | Yes | No |
| * ASD groups showed smaller costs than healthy controls in these studies | | | |

**Supplementary Material B.**

This section contains a description and schematic of the tasks administered in our own lab that are reported in Table 1 in the main text. Note that timing and exact formats may differ.

***Eriksen flanker task.*** Participants responded to the direction of a centrally presented arrow (left or right) using the \ and / keys. On each trial, the centrally presented arrow (1cm x 1cm) was flanked above and below by two other symbols separated by 0.75cm. Flanking stimuli were either arrows pointing in the same direction as the central arrow (congruent condition), straight lines (neutral condition), or arrows pointing in the opposite direction to the central arrow (congruent condition). Stimuli were presented until a response was given, with an Inter-Stimulus Interval (ISI) of 750ms.

***Stroop task.*** Participants responded to the colour of a centrally presented word (Arial, font size 70), which could either be red (z key), blue (x key), green (n key) or yellow (m key). The presented word could be the same as the font colour (congruent condition), one of four non-colour words (lot, ship, cross, advice) taken from Friedman and Miyake (2004) matched for length and frequency (neutral condition), or a colour word corresponding to one of the other response options (incongruent). Stimuli were presented until a response was given, with an ISI of 750ms.

***Simon task.*** Participants responded to the colour of a circle (2cm in diameter), which was either blue (\ key) or green (/ key). The circle could be presented either centrally (neutral condition), or 4cm to the left or right of centre, making them either congruent or incongruent with correct response hand. Stimuli were presented until a response was given, with an ISI of 750ms.

**Spatial-numerical association of response codes (SNARC) task.** Participants were required to determine whether a centrally presented white digit (1 to 9, excluding 5; Arial, font size 70) was greater or less than five using the z and m keys. The response mapping alternated across blocks, with the first block being counter-balanced across participants. Participants were informed of the response mapping before each block and eight ‘buffer’ trials were presented at the start of each block to accommodate the change in response rules. (Rusconi et al., 2013). These buffer trials were subsequently discarded for analysis. Participants were also presented with feedback if they gave an incorrect response, lasting 1000ms.

**Navon task.** Participants were presented with composite letter stimuli; large ‘H’ or ‘S’ characters (3cm x 4.5cm) comprised of smaller ‘S’ or ‘H’ (0.4cm x 0.7cm) characters. Stimuli could either be consistent, in which the same character appeared at the global and local levels, or inconsistent (e.g. a large H composed of smaller S characters). Stimuli were presented at one of four possible locations, 0.5cm above or below and 2cm to the left or right of fixation. Before each block, participants were instructed that they were to respond to either the global or local character. The relevant property of the stimulus alternated across blocks, and was counter-balanced across participants. Participants were presented with eight buffer trials at the beginning of each block, and received feedback if they gave an incorrect response.

***Antisaccade task.*** The antisaccade task consisted of both prosaccade and antisaccade trials presented in separate blocks. The prosaccade task began with the initial fixation screen for 700ms presented at 10cd/m^2^ and occupying 0.1 deg^2^, and two place-markers, each presented at 10cd/m^2^ and 8 degrees eccentricity. Placemarkers consisted of 8mm^2^ square-frames. All stimuli were presented against a light grey background of 25cd/m^2^. Saccades were directed to a target which obscured either the left or right placemarker (direction was determined randomly). The target consisted of a small black square, 0.1 deg^2^ in size and presented at 1cd/m^2^ and 8 degrees eccentricity. On antisaccade trials, participants were instructed to saccade to the opposite placemarker from an anti-target which was a large light-grey square, 30mm^2^ in size and presented at 30cd/m^2^. The square was presented such that its centre lay at 8 degrees eccentricity. Targets were displayed for 500ms, followed by a 500ms blank screen.

***Remote Distractor task.*** The target in the remote distractor task was the same stimulus as was used as the prosaccade target (a small black square) and the distractor stimulus (to be ignored by the participant) was the same as was used as the anti-target (a large light-grey square). After 700ms fixation, the target appeared on either the left or the right of the screen for 500ms. On distractor trials, the distractor appeared simultaneously on the opposite side to the target. Target presentation was followed by a blank screen for 500ms. Distractor and prosaccade trials were randomly intermixed within blocks.

***Illogical rule task.*** Logical and illogical rule trials were presented in separate blocks. At the start of each trial, a red and blue square appeared on either side of fixation. After 700ms the fixation cross turned either red or blue for 500ms, followed by a blank screen for 500ms. In the logical rule blocks, participants were instructed to saccade to the target corresponding to the colour of the fixation cross. The illogical task used the same stimuli as the logical rule, but participants were instructed to saccade to the stimulus that did not correspond to the colour of the fixation cross (i.e. if the cross turned red, they were to saccade to the blue target).


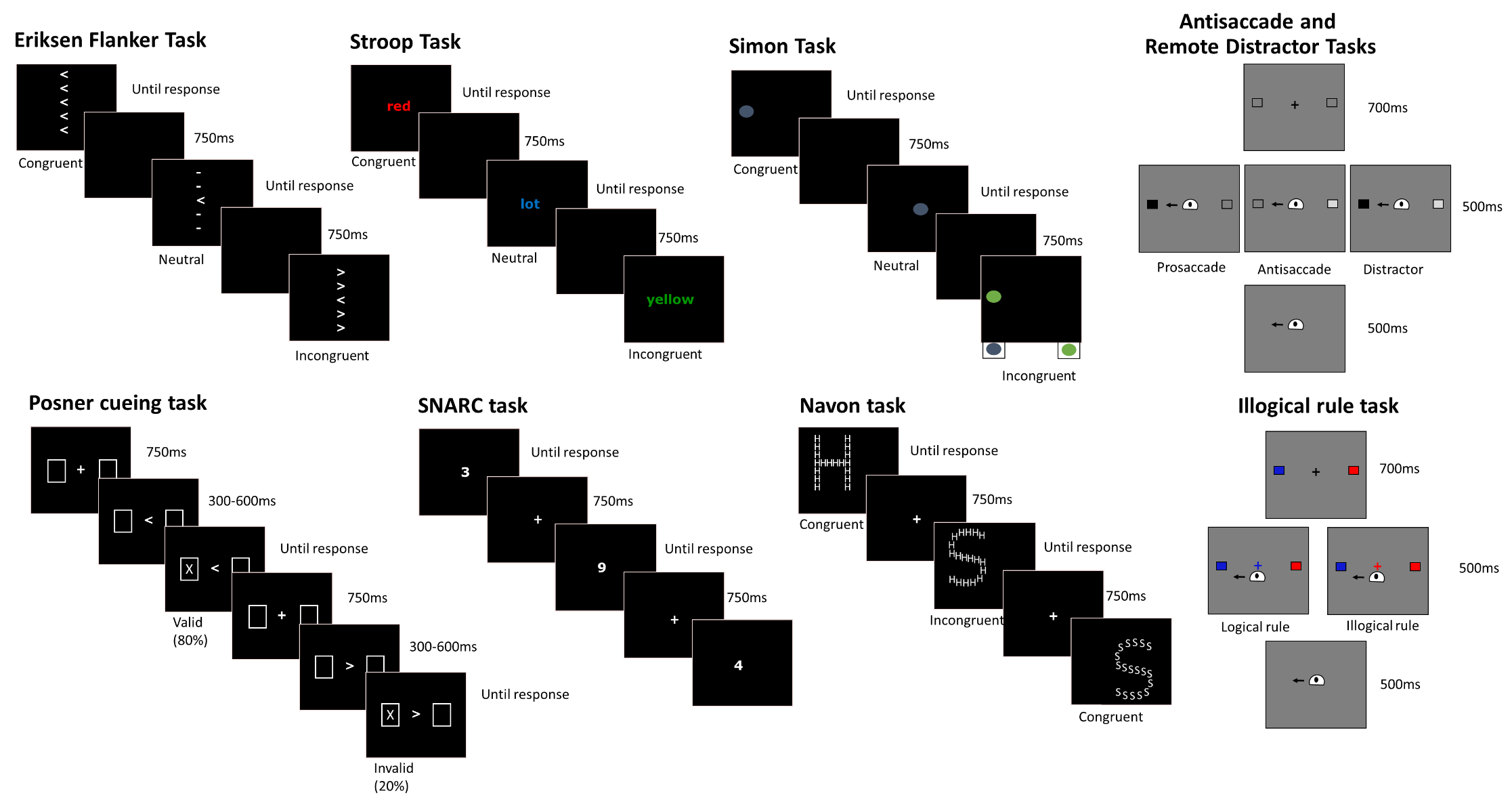


Supplementary Figure B1. Schematic of tasks used and their conditions.

**Supplementary Material C.**

This section contains additional information to that contained in Table 1 in the main text. We report the sample from which the dataset was obtained, in addition to comments on how data were selected in order to replicate the correlations reported in Table 1. For full methodological details on each task, we refer readers to the original papers.

For correlations in one paper (Manoach et al., 2002), we reversed the sign of the correlation reported in text so the direction of the error cost was consistent with our other datasets. We did this because another paper from the same research group (Cherkasova et al., 2002), for which we extracted the data from a plot (their Figure 4) and calculated the correlation ourselves, computed the accuracy cost as Switch accuracy – Repetition accuracy. This produces an error cost in the opposite direction to how our other effects are coded, such that the sign of the correlation coefficient needed to be reversed for our meta-analysis. Correlations from both papers are reported in Barton et al. (2002), so we assumed the*y* were calculated consistently.

Table C1. Study information for datasets used to calculate correlations between reaction time costs and error costs in cognitive task (see Table 1 in main text). Unpublished data refer to data collected in our own laboratory, in part to address this topic. New analysis of published data refer to analyses conducted by us of published datasets that we were able to obtain (see methods). These correlations were not reported in the original papers. Previously reported correlations refer to correlations included in published papers, for which we conducted no additional analyses.

|  | Study | Task/effect | N | Trial N (baseline/alternate) | Sample | Data selection |
| --- | --- | --- | --- | --- | --- | --- |
| Our data and unpublished data | Hedge et al. (2017) | Flanker | 104 | 480/480 | University students | Data are pooled across two sessions occurring three weeks apart. Flanker and Stroop datasets combines two separate studies with identical procedures. |
|  |  | Stroop | 103 | 480/480 |  |  |
|  |  | SNARC | 40 | 640/640 |  |  |
|  |  | Navon – local conflict | 40 | 320/320 |  |  |
|  |  | Navon – global conflict | 40 | 320/320 |  |  |
|  |  | Navon – global precedence | 40 | 320/320 |  |  |
|  | Hedge et al. (In prep) | Flanker | 50 | 336/336 | University students |  |
|  |  | Simon | 50 | 336/336 |  |  |
|  | Other data | Antisaccade | 48 | 200\|400/300\|400† | University students | Data are pooled across two sessions occurring one week apart. |
|  |  | Illogical rule task | 44 | 200/200 |  |  |
|  |  | Distractor | 48 | 200/200 |  |  |
|  |  | Antisaccade | 21 | 400/400 | University students |  |
| New analysis of published data | *Aichert et al. (2012)* | Antisaccade | 502 | 60/60 | General community | **Data obtained from author.** |
|  | Balota et al. (2007) | Lexical Decision task (English) | 809 | 1686/1686†† | University students or participant panel | **Source: http://elexicon.wustl.edu/**  Data are pooled across two sessions. |
|  | Braem (2017) | Task switching | 49 | 78/78 | University students | **Source: https://osf.io/h3e7w/** |
|  | Bugg and Braver (2015) | Exp 1-Task switching | 52 | 185/199† | University students | **Source: https://osf.io/fveqb/** |
|  |  | Exp 1-Rule congruency | 52 | 192/192 |  |  |
|  |  | Exp 1-List congruency | 52 | 192/192 |  |  |
|  |  | Exp 2-Task switching | 32 | 225/223† |  |  |
|  |  | Exp 2-Rule congruency | 32 | 224/224 |  |  |
|  |  | Exp 2-List congruency | 32 | 224/224 |  |  |
|  |  | Exp 3-Task switching | 32 | 123/131†† |  | Data are taken from mixed trial blocks only |
|  |  | Exp 3-Rule congruency | 32 | 380/126†† |  |  |
|  |  | Exp 3-Incentive | 32 | 46/47†† |  | Data includes only congruent repeat trials in incentive blocks |
|  |  | Exp 3-Mixed task | 32 | 252/123 |  | Data excludes switch trials (only present in mixed task blocks) |
|  | *Chen et al. (2015)* | Flanker | 42 | 120/120 | University students | **Source: https://figshare.com/articles/VBM_Eriksenflanker/1483531** |
|  | *Cherkasova et al. (2002)* | Task-switch (antisaccade) - controls | 18 | 104/104 | 13 to 54 years (mean 30.8 years, SD = 9.5). | Data were extracted from Figure 4 (pg 534) using webplotdigitizer (https://automeris.io/WebPlotDigitizer/). Sign of correlation was reversed (see text above) |
|  | *Chetverikov et al. (2017)* | Flanker (colour) | 58 | 120/120 | University students | **Source: https://osf.io/mtza2/** |
|  | De Simoni & Von Bastian (2018) | Simon | 216 | 192/192 | University students | **Source: https://osf.io/fy5ku/** Data are collapsed across two sessions taking place five weeks apart. |
|  |  | Stroop | 216 | 192/192 |  |  |
|  |  | Numeric Stroop | 216 | 192/192 |  |  |
|  |  | Navon (conflict) | 216 | 192/192 |  |  |
|  |  | Task switching (animacy/size) | 216 | 128/128 |  |  |
|  |  | Task switching (shape/colour) | 216 | 128/128 |  |  |
|  |  | Task switching (parity/magnitude) | 216 | 128/128 |  |  |
|  |  | Task switching (fill/frame) | 216 | 128/128 |  |  |
|  |  | Task mixing (animacy/size) | 216 | 512/128 |  | Data excludes switch trials (only present in mixed task blocks) |
|  |  | Task mixing (shape/colour) | 216 | 512/128 |  |  |
|  |  | Task mixing (parity/magnitude) | 216 | 512/128 |  |  |
|  |  | Task mixing (fill/frame) | 216 | 512/128 |  |  |
|  | Ebersole et al. (2016) | Stroop | 3305 | 21/42 | University students and online | **Source: https://osf.io/ct89g/** |
|  | Elchlepp et al. (2017) | Task-switch | 21 | 878/438†† | University students | **Source: https://ore.exeter.ac.uk/repository/handle/10871/24754** |
|  | Ferrand et al. (2010) | Lexical decision task (French) | 868 | 1000/1000 | University students | **Source: https://link.springer.com/article/10.3758/BRM.42.2.488#SupplementaryMaterial** |
|  | Gonthier et al. (2016) | Stroop (picture/word) | 95 | 600/600 | University students & general community | **Source: https://osf.io/b9zyv/** |
|  | Guye & Von Bastian (2017) | Flanker | 142 | 192/192 | Older adults (range: 65–80) | **Source: https://osf.io/zrj3q/** Data are collapsed across two sessions taking place five weeks apart. |
|  |  | Simon | 142 | 192/192 |  |  |
|  |  | Stroop | 142 | 192/192 |  |  |
|  |  | Task switching (animacy/size) | 142 | 128/128 |  |  |
|  |  | Task switching (shape/colour) | 142 | 128/128 |  |  |
|  |  | Task switching (parity/magnitude) | 142 | 128/128 |  |  |
|  |  | Single vs. mixed task (animacy/size) | 142 | 512/128 |  | Data excludes switch trials (only present in mixed task blocks) |
|  |  | Single vs. mixed task (shape/colour) | 142 | 512/128 |  |  |
|  |  | Single vs. mixed task (parity/magnitude) | 142 | 512/128 |  |  |
|  | Hefer et al. (2017) | Flanker | 73 | 60/60 | University students | **Source: https://epub.uni-regensburg.de/35799/** Data excludes trials on which a prospective memory cue was presented |
|  |  | Flanker | 64 | 72/72 |  |  |
|  | Kelly et al. (2008) | Flanker | 26 | 24/24 | Healthy adults | **Source: https://openneuro.org/datasets/ds000102** |
|  | Keuleers et al. (2010) | Lexical decision task (Dutch) | 39 | 14089/14089 | University students and employees | **Source: http://crr.ugent.be/dlp.** Data are collapsed across multiple sessions. |
|  | Keuleers et al. (2012) | Lexical decision task (English) | 79 | 14365/14365 | University students and employees | **Source: http://crr.ugent.be/blp** Data are collapsed across multiple sessions. |
|  | *Klein et al. (2017)* | *Stroop* | *276* | *80/80* | *University students* | **Source: https://osf.io/xq9mu/** *Data are combined from Experiments 1 and 2* |
|  | *Klemen et al.(2011)* | Flanker | 18 | 48/48 | University students | **Data obtained from author.** are taken from uncued stimuli at 0 SOA in their expeirment 1. |
|  | *Kreitz et al. (2015)* | Flanker | 120 | 50/50 | University students | **Source: https://osf.io/mvwih/** Additional information obtained from author |
|  |  | Flanker | 197 | 50/50 |  |  |
|  | Mennes et al. (2012) | Simon | 21 | 96/96 | Healthy adults | **Source: https://openneuro.org/datasets/ds000101/versions/00004** |
|  | Peronne-Bertolotti et al. (2017) | Manual 'antisaccade' | 44 | 256/256 | University students | **Source: http://journals.plos.org/plosone/article?id=10.1371/journal.pone.0180084#sec028** Data are collapsed across a gaze cueing manipulation |
|  |  | Task mixing | 44 | 256/256 |  |  |
|  | *Rusconi et al. (2013)* | SNARC | 17 | 56/56 |  | **Data obtained from author.** Study featured transcranial magnetic stimulation - only sham condition used here |
|  | Sandra and Otto (2018) | Stroop | 57 | 90/30 | University students and participant panel | **Source: https://osf.io/gqvsh/** |
|  |  | Task switching | 62 | 130/148†† |  |  |
|  |  | Reward | 62 | 140/140 |  |  |
|  | Saunders et al. (2015) | Flanker | 56 | 250/250 | University students | **Source: osf.io/mtrys** Two datasets come from same participants pre- and post- mood induction. Two participants' data are omitted from the pre- dataset, so we include them separately. |
|  |  | Flanker | 58 | 250/250 |  |  |
|  | *Saunders et al. (2018)* | Stroop | 217 | 288/288 | University students | **Source: https://osf.io/8etus** |
|  |  | Flanker | 2249 | 50/50 | Online sample | Data are combined across experiments 2a - 2c |
|  | Von Bastian et al. (2016) | Flanker | 120 | 48/48 | University students | **Data obtained from author** |
|  |  | Simon | 120 | 150/50 |  |  |
|  |  | Numerical Stroop | 120 | 48/48 |  |  |
|  |  | Task switching (animacy/size) | 120 | 64/64 |  |  |
|  |  | Task switching (colour/shape) | 120 | 64/64 |  |  |
|  |  | Task switching (parity/size) | 120 | 64/64 |  |  |
|  |  | Single vs. mixed task (animacy/size) | 120 | 256/64 |  | Data excludes switch trials (only present in mixed task blocks) |
|  |  | Single vs. mixed task (colour/shape) | 120 | 256/64 |  |  |
|  |  | Single vs. mixed task (parity/size) | 120 | 256/64 |  |  |
|  | Wöstmann et al. (2013) | Flanker | 23 | 80/80 | General community | **Data obtained from author.** Data are collapsed across sessions |
|  |  | Simon | 23 | 320/120 |  |  |
|  | Xu et al. (2014) | Age Implicit Association Test (IAT) | 981873 | 40/40 | Online sample | **Source: https://osf.io/y9hiq/** |
|  |  | Arab IAT | 338103 | 40/40 |  |  |
|  |  | Asian IAT | 374882 | 40/40 |  |  |
|  |  | Disability IAT | 309792 | 40/40 |  |  |
|  |  | Gender-Career IAT | 852861 | 40/40 |  |  |
|  |  | Gender-Science IAT | 636003 | 40/40 |  |  |
|  |  | Native American IAT | 217444 | 40/40 |  |  |
|  |  | President IAT | 379465 | 40/40 |  |  |
|  |  | Race IAT | 3339097 | 40/40 |  |  |
|  |  | Religion IAT | 169247 | 40/40 |  |  |
|  |  | Sexuality IAT | 1452795 | 40/40 |  |  |
|  |  | Skin colour IAT | 872781 | 40/40 |  |  |
|  |  | Weapons IAT | 534563 | 40/40 |  |  |
|  |  | Weight IAT | 969372 | 40/40 |  |  |
|  | Zwann et al. (2017) | Flanker | 160 | 64/64 | Online sample | **Source: https://osf.io/ghv6m/** |
|  |  | Simon | 160 | 92/92 |  |  |
| Previously reported correlations | Manoach et al (2002) | Task-switch (antisaccade) -schizophrenia | 21 | 104/104 | Schizophrenia patients | Sign of correlations are reversed from those stated in text - see text above table for explanation. |
|  |  | Task-switch (antisaccade) - controls | 16 | 104/104 | Matched controls for Schizophrenia patients |  |
|  | Draheim et al. (2016) | Task switching | 552 | 96/96 | University students and general community | Correlation reported in Table 3 (pg. 142). Trial number information obtained from author. |
|  | Hughes et al. (2014) | Task switching | 1902 | 46/98 | US military trainees | Correlations reported in their Table 5 (pg. 716). Trial numbers confirmed with author. |
|  |  | Task switching | 46 | 264/120 | University students |  |
|  | Kane and Engle (2003) | Stroop | 87 | 36/36 | University students | Correlations reported in text on page 65 |
|  |  | Stroop | 88 | 36/36 |  |  |
|  |  | Stroop | 138 | 36/36 |  |  |
|  | MacLeod et al. (2010) | Attention Networks Test – Alerting | 1129 | 72/72 | Healthy adults - combines several datasets | Correlations reported in figure 5 (pg. 645) |
|  |  | Attention Networks Test – Orienting | 1129 | 72/72 |  |  |
|  |  | Attention Networks Test: Executive | 1129 | 96/96 |  |  |
|  | Paap and Sawi (2014) | Manual "antisaccade" | 117 | 30/60 | University students | Correlation reported in Table 7 (pg 12) |
|  | Rondeel et al. (2015) | Stroop | 35 | 54/54 | University students | Correlation reported in Table 3 (pg 8). |
|  | Wylie et al. (2009) | Flanker (Parkinsons patients) | 50 | 103/103 | Parkinsons disease patients | Correlation reported in text on pg 149. Correlation not reported for healthy controls, though noted to be not negative |

**Supplementary Material D.**

Figure 4 in the main text gives an outline of how high and low response caution and response selection relate to RT costs and error costs in the context of the drift-diffusion model. Here, we show the equivalent plots for the LBA and ALIGATOR models. The behaviour of the DMC is similar to the standard DDM in this context, so we do not plot this separately.


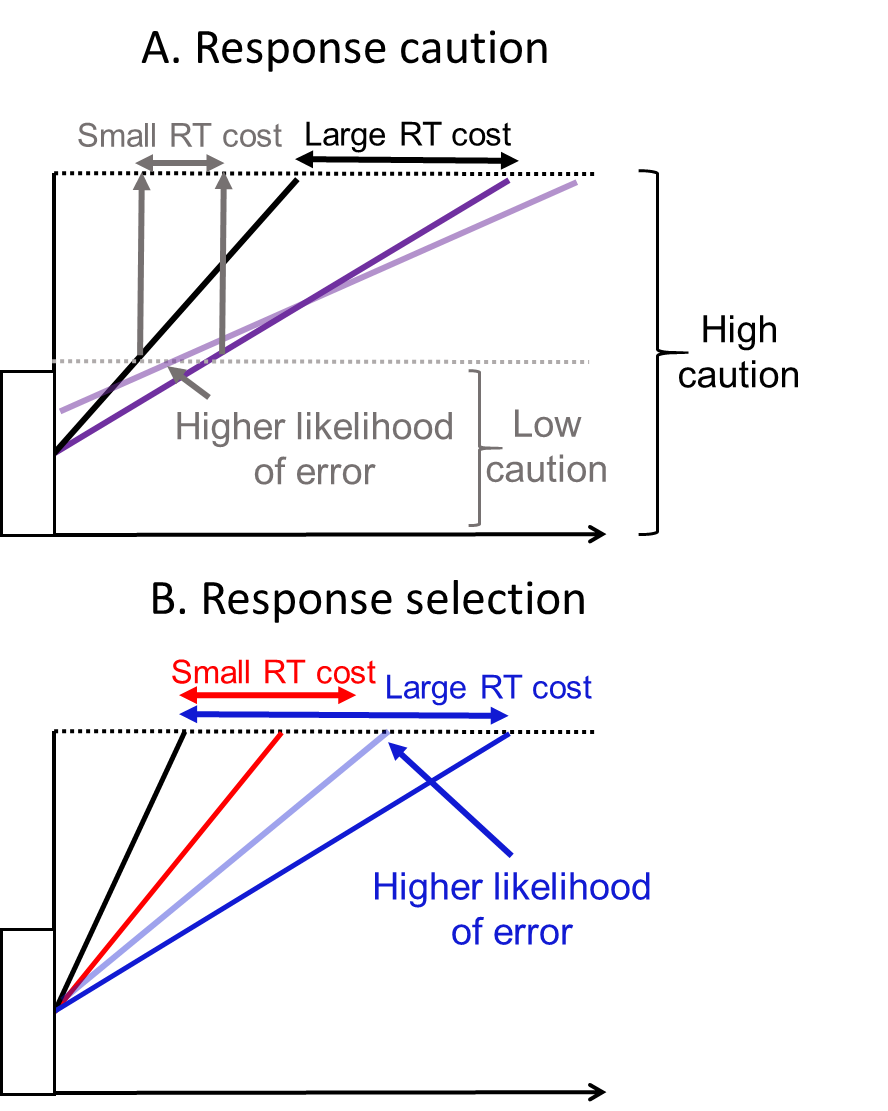


*Figure D1.* Pattern of RT costs and Error costs produced by variation in response caution and selection in the linear ballistic accumulator model. Straight, solid lines show condition averages, faint lines show example individual trials. Black lines show drift rates in congruent/baseline condition, coloured lines show incongruent condition. A. Response caution: Individuals who are low in response caution will set a lower threshold (e.g. grey dotted line) than highly cautious individuals (black dotted line). This means not only that their RTs will be faster, but also the difference between conditions will be smaller, leading to smaller RT costs, noted by grey arrows compared to black arrows. However, the lower threshold will lead to more errors due to variability in start points, which can be overcome with higher thresholds (example trial in purple reaches the grey error threshold, but not the black error threshold). Note that this will predominantly affect the incongruent or more difficult condition, as errors are rare in congruent/baseline conditions, leading to higher relative error costs. B. Response selection: Individuals who have high selection efficiency will have relatively higher drift rates in incongruent conditions (red solid lines) compared to individuals with lower selection efficiency (blue solid lines), leading to smaller RT costs (noted by red arrows compared to blue arrows). Moreover, the higher drift rate means noise is less likely to cause the incorrect response (illustrated with blue example trial that reaches the error threshold).

*
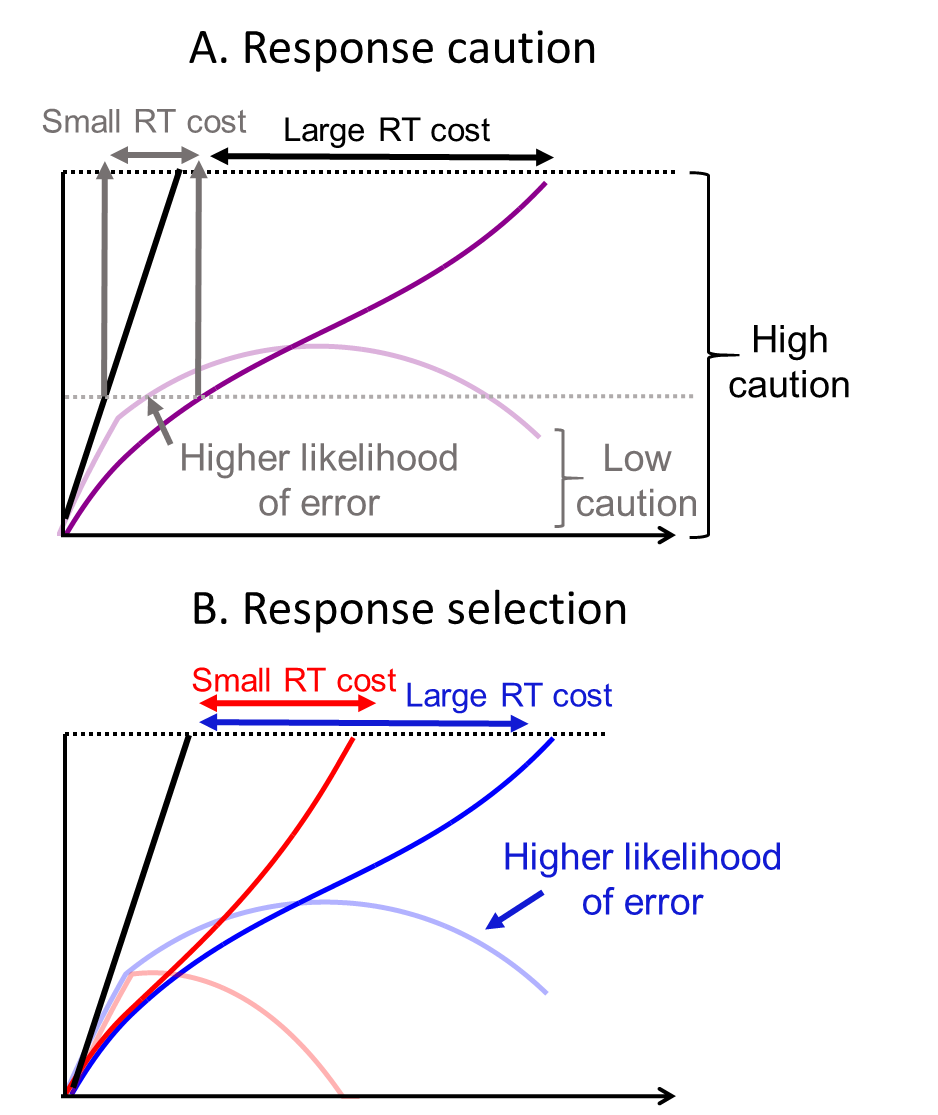
*

*Figure D2. Pattern of RT costs and Error costs produced by variation in response caution and response selection in ALIGATER. Solid lines and dashed lines show condition averages, dotted lines show example individual trials. A. Response caution: Individuals who are low in response caution will set a lower threshold (grey dotted line) than highly cautious individuals (black dotted line). This means not only that their RTs will be faster, but also the difference between conditions will be smaller, leading to smaller RT costs (noted by grey arrows compared to black arrows). However, variability in drift rates will mean that some trials will produce errors (purple dotted line), which can be overcome with higher thresholds. B. Response selection: Individuals who have high selection efficiency will have relatively reactive inhibition in incongruent conditions (red solid lines) compared to individuals with lower selection efficiency (blue solid lines), leading to smaller RT costs (noted by red arrows compared to blue arrows). Moreover, the higher level of reactive inhibition strength means that the accumulator for the error response is less likely to reach threshold. The red and blue dotted lines show the accumulators for the error responses on a single trial with identical drift rates. The red dotted line (higher selection efficiency) is inhibited faster than the blue dotted line (lower selection efficiency).*

**Supplementary Material E**

This section contains detailed methods and descriptive results for Studies 1 and 2.

**Method**

**Participants.**

Participants were 57 (Study 1; 6 male) and 81 (Study 2; 6 male) undergraduate and postgraduate psychology students. Participants took part either for payment or for course credit. All participants gave their informed written consent prior to participation in accordance with the revised Declarations of Helsinki (2013), and the experiments were approved by the local Ethics Committee.

**Design and Procedure.**

***Study 1.*** Participants completed both the Stroop and flanker task in two 90 minute sessions taking place approximately 4 weeks apart. The format of these tasks was as described in Supplementary material B, with the following exceptions.

For each session and task, participants completed 12 blocks, consisting of 4 each for speed, standard and accuracy instructions. Each block consisted of 144 trials, with 48 each of congruent, neutral and incongruent stimuli (192 trials total per congruency and instruction condition). The order of blocks was randomised, as was the order of trials within blocks. At the beginning of speed-emphasis blocks, participants were asked to “Please try to respond as quickly as possible, without guessing the response”. For accuracy blocks, participants were told “Please ensure that your responses are accurate, without losing too much speed”. For standard instruction blocks, participants were instructed “Please try to be both fast and accurate in your responses”. In speed blocks, if participants responded slower than 500ms in the flanker or 600ms in the Stroop (based approximately on typical response time distributions for these tasks in our lab), the message “Too slow” appeared on screen for 500ms. In the accuracy condition, the message “Incorrect” appeared if participants made an error. In all blocks, the message “Too fast” appeared if participants responded faster than 150ms in the flanker and 200ms in the Stroop task (typically <1% of trials). Participants received feedback about both their average RT and accuracy after each block in all instruction conditions.

***Study 2.*** Participants completed both the flanker task and a dot motion discrimination task based on Pote et al. (2016). The flanker task appeared as described above. Participants performed 12 blocks of 144 trials in total. Twelve participants did not complete the task within the allotted time, so data were only available for 11 (11 participants) or 10 (1 participant) blocks.

In the dot motion task, each frame consisted of 50 white dots (5x5 pixels in size) displayed within an oval patch (14.7cm high x 23.7cm wide) in the centre of a grey screen (60hz, 1680x1050). On each frame, either 30% (high coherence) or 15% (low coherence) of the dots were chosen as signal dots, which moved in a consistent direction (left or right) by 29 pixels. The lifetime of the dots was 3 frames. Non-signal dots reappeared in a random position on each frame. The stimulus was displayed for a maximum of 2000ms, with a 500ms ISI. Participants were asked to determine the direction of the coherent motion. Each block consisted of 120 trials, 60 of each coherence level. Participants performed 12 blocks in total, except for 5 participants who completed 11 blocks, and 1 participant who completed 10.

Feedback relating to speed, accuracy or neutral blocks was given as described in Study 1. For the dot motion task, participants were informed that their responses were too slow in speed blocks if their RT exceeded 700ms. Participants were informed that they were too fast in all blocks if their responses were shorter than 250ms.

**Data analysis.** Two participants from Study 1 were removed because they did not complete the session. The same inclusion criteria and RT cut-offs described in Part 1 were applied; the number of participants included in the analysis is shown in Table 3.

**Descriptive results**

This section contains the means and standard deviations (in parentheses) for the data in Studies 1 and 2 (Part 2 – see Table 3 in main text). In that section, we note that increased correlations between RT costs and error costs are not systematically determined by increases or decreases in the variance of the measures. The variance of the RT costs is typically higher (or similar) under standard instructions, whereas the variance of the error costs is higher under speed instructions. Therefore, it is not that participants are more variable overall in one instruction condition.

Similar to the pattern of variances, the mean RT costs are smaller under speed compared to standard instructions, whereas the mean error cost is increased. This reflects participants’ compliance with the instructions. RTs overall are faster under speed instructions, such that the difference between conditions is smaller, and the error cost increased. This is analogous to the simulation of a low and high caution individual in Figure 2.

Table E1. Mean RT costs (ms) and error costs (%) for studies 1 and 2. Standard deviations are reported in parentheses.

|  | Speed instructions | | Standard instructions | | Accuracy instructions | |
| --- | --- | --- | --- | --- | --- | --- |
| Dataset | RT cost | Error cost | RT cost | Error cost | RT cost | Error cost |
| Flanker 1 Session 1 | 24 (19) | 17.2 (8.5) | 37 (20) | 12.8 (6.6) | 44 (18) | 9.4 (5.4) |
| Flanker 1 Session 2 | 17 (16) | 14.5 (7.9) | 37 (18) | 9.3 (5.4) | 37 (18) | 9.3 (5.4) |
| Stroop 1 Session 1 | 18 (21) | 6.8 (5.4) | 69 (41) | 4.5 (4.4) | 74 (42) | 4.1 (3.3) |
| Stroop 1 Session 2 | 12 (21) | 7.6 (6) | 53 (39) | 5.9 (4.8) | 63 (37) | 3.6 (4.7) |
| Flanker 2 | 18 (21) | 12 (8.5) | 32 (22) | 10.9 (6.4) | 37 (22) | 8.9 (6.3) |
| Dot-motion 2 | 9 (13) | 9.7 (5.2) | 34 (43) | 13.1 (4.2) | 46 (36) | 13.5 (3.7) |

**Supplementary Material F**

This section contains detailed methods for Study 3.

**Participants.** Participants were 102 undergraduate psychology students (12 male). Participants took part for course credit. All participants gave their informed written consent prior to participation in accordance with the revised Declarations of Helsinki (2013), and the experiments were approved by the local Ethics Committee.

**Design and Procedure.** Participants completed a Simon task, the stimuli for which appeared as described in Part 1. There were 8 blocks, each containing 144 trials. Half of these blocks contained an equal number of congruent and incongruent trials randomly intermixed, while two blocks each were comprised solely of either congruent or incongruent trials. This produced a total of 288 trials per congruency condition per block type. The blocks were alternated throughout the session in a consistent order (mixed, congruent only, mixed, incongruent only), with the starting block counterbalanced across participants using a latin square. As we are interested in the difference between correlations between blocking conditions, we wanted to avoid lower (or higher) correlations being produced in certain conditions due to order effects (e.g. fatigue). Participants were informed of the composition of each block before starting each one.

Participants initially completed eight practice trials in which they received trial by trial feedback about the accuracy of their response. During the test phase, they only received feedback about their average error rates and RT at the end of each block. Participants were tested in groups of up to eight, at separate workstations separated by dividers. An experimenter was present throughout to monitor compliance.

**Data analysis.** The same inclusion criteria and RT cut-offs described in Part 1 were applied; all participants were retained for analysis.

**Supplementary Material G**

In this section, we simulate the impact of varying other parameters of potential interest on the RT cost – error cost correlation. We discuss the implications of these in the general discussion of the main text.

In the first set of simulations, we vary other parameters in the drift-diffusion model to reflect three scenarios. The parameters used to simulate these data are shown in Table G1. These are based on the simulation ranges used in our main analysis (see Table 2 and Figure 6 in the main text). The results of these simulations are shown in Figure G1. We reproduce the original DDM simulation results in the top row for comparison.

**Average drift rate/general slowing scenario (2^nd^ row)**

In this scenario we hold the difference between drift rates for congruent and incongruent trials constant (.2), and vary the average drift rate from .2 to .5 in increments of .05. Lower average drift rates produce increased error costs and increased RT costs, leading to a positive correlation between the two. In other words, general slowing could mimic the effects of a domain specific deficit. Lower average drift rates also produces slower RTs and increased errors in both conditions.

**Non-decision time (3^rd^ row)**

In this scenario we hold all the decision parameters (drift rates and boundary separation) constant, as well as holding non-decision time constant in the incongruent condition (500ms). We vary non-decision time in the congruent condition from 500ms to 200ms. Note that we plot the difference between non-decision times on the x-axis in the first two columns. Increasing the difference between non-decision times in each condition increases the RT cost while having no effect on the error cost, leading to no correlation.

**Starting point variability (4^th^ row)**

In this set of simulations, we vary starting point variability (sz) from .0805 to .0115, which we express as a proportion of boundary separation (.1 to .7). The values describe the range of a uniform distribution centred on the average (unbiased) starting point z. High levels of starting point variability reflects a larger number of trial in which the accumulation process begins close to the boundary. Generally, this parameter has little impact on the RT cost – error cost correlation, as it leads to an increase in both fast error and fast correction responses. At higher levels, a slight increase in the error cost is observed.

Table G1. Parameter ranges used in first set of drift-diffusion model simulations (see Figure G1 and text).

|  | Incongruent drift rate | Boundary separation | Congruent drift rate | Variability in drift rate | Start point bias | Within-trial noise | Non-decision time congruent | Non-decision time incongruent | Starting point variability/ boundary separation |
| --- | --- | --- | --- | --- | --- | --- | --- | --- | --- |
| General slowing | .1 - .4 | 0.115 | .3 - .6 | 0.1 | 0.5 | 0.1 | - | - | - |
| Non-decision time | 0.25 | 0.115 | 0.45 | 0.1 | 0.5 | 0.1 | 200 - 500 | 500 | - |
| Starting point variability | 0.25 | 0.115 | 0.45 | 0.1 | 0.5 | 0.1 | - | - | .1-.7 |


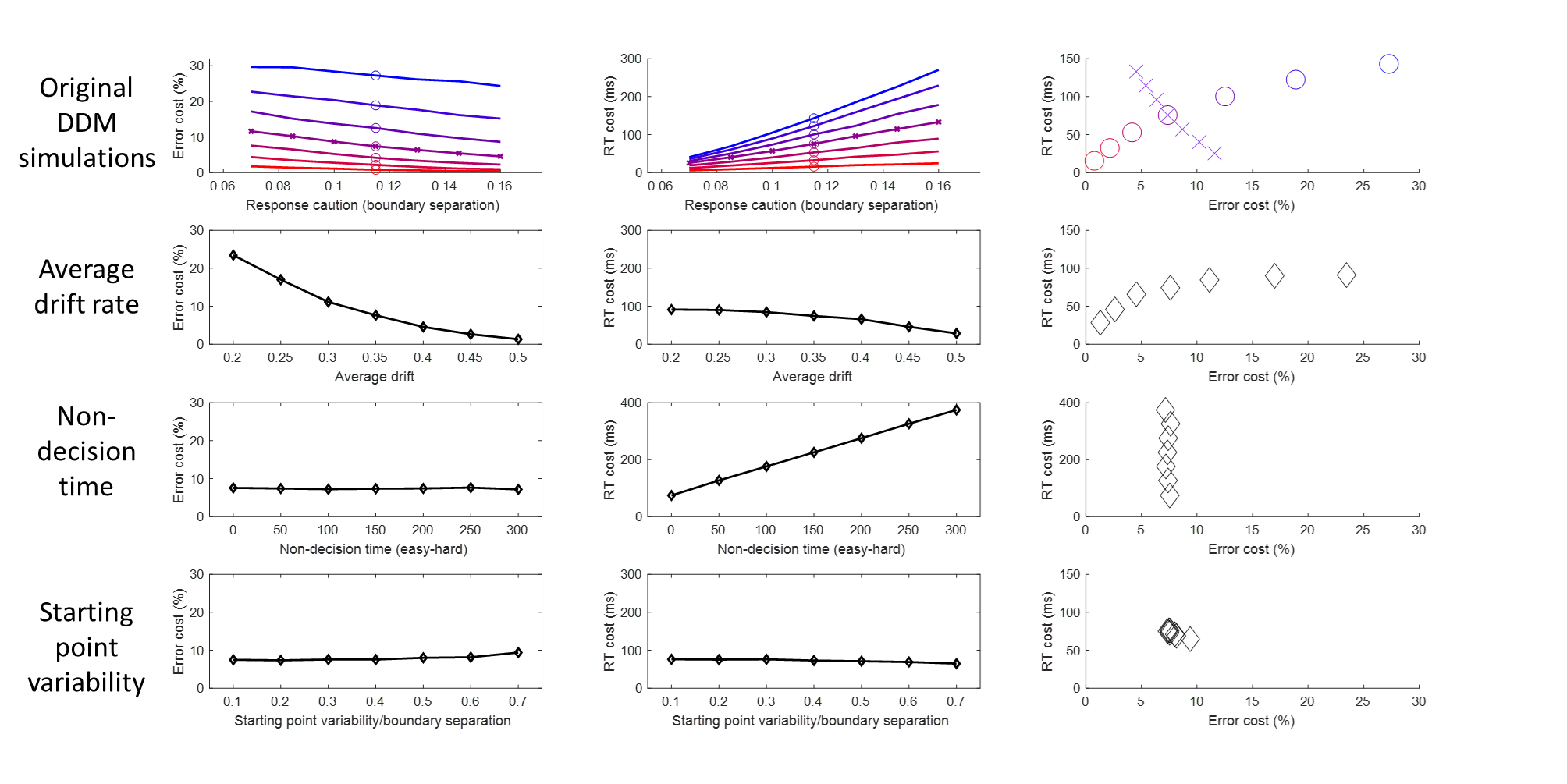
Figure G1. Simulated error costs and RT costs produced by drift-diffusion model under four scenarios. The top row reproduces the results from our DDM simulations in the main text (See Figure 6). Variation in response caution is shown on the x-axis. Variation in response selection is indicated by different lines, ranging from low selection ability (blue) to high selection ability (red). The first and second columns show the patterns of error costs and RT costs, respectively, as a function of variation in both caution and response selection as implemented in the different models (see main text for details). The third column shows the correlation between RT costs and error costs that arise from holding response selection constant and allowing caution to vary (purple line and crosses), and for allowing response selection to vary while caution is held constant (grey line and circles). The second to forth rows show the effect on RT costs and error costs where response selection and response caution are held constant, and another parameter is varied (x-axis) according to each scenario. See text for further details.

**Time-to-peak of the automatic activation (diffusion model for conflict tasks)**

In our second set of simulations, we examine the impact of varying the time-to-peak parameter in the diffusion model for conflict tasks. The time-to-peak parameter affects the time course of automatic activation in the model, which accounts pre-potent response activation in response conflict tasks (e.g. the flanker and Simon tasks). A shorter time-to-peak means that the automatic activation initially rises quickly, but is also quickly suppressed. A short time-to-peak is necessary to account for negative going delta functions in the Simon task; that is, the RT cost is reduced in slow RTs.

The relevance for our discussion is that shorter time-to-peak values may lead to a reduction in the RT cost, confounding what would otherwise be a positive correlation between RT costs and error costs. In our DMC simulations in the main text, we fix the time-to-peak parameter to 90, and observed a positive correlation between RT costs and error costs when varying the amplitude of the automatic activation parameter (A). In their original paper, Ulrich et al. (2015) observed an average time-to-peak of 118ms for the flanker task, and 35ms for the Simon task. In this new simulation, we fix the other parameters in the DMC to the averages used in our main simulation (A = 19, Boundary separation = 50; drift rate = .63, starting point shape = 2), and vary the time-to-peak parameter from 25 to 145 in intervals of 20.

The results are show in Figure G2 (second row). We reproduce our original DMC simulations in the first row for comparison. Earlier time to peak values lead to an increase in error costs as fast errors in incongruent trials increase. Very early time to peak values also lead to a slight reduction in the RT cost (due to negative going delta slopes). However, note that this primarily occurs below the average previously reported for the Simon task (35ms); higher time-to-peak values would be expected for other conflict tasks.


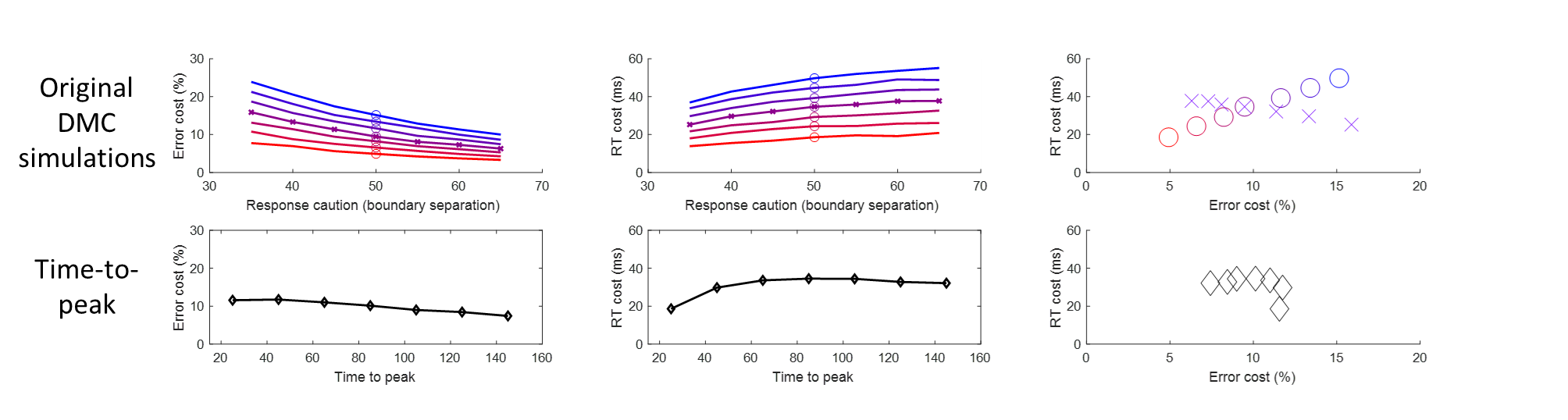


Figure G2. Simulated error costs and RT costs produced by diffusion model for conflict tasks. The top row reproduces the results from our DMC simulations in the main text (See Figure 6). Variation in response caution is shown on the x-axis. Variation in response selection (the amplitude of automatic activation) is indicated by different lines, ranging from low selection ability (blue) to high selection ability (red). The first and second columns show the patterns of error costs and RT costs, respectively, as a function of variation in both caution and response selection as implemented in the different models (see main text for details). The third column shows the correlation between RT costs and error costs that arise from holding response selection constant and allowing caution to vary (purple line and crosses), and for allowing response selection to vary while caution is held constant (grey line and circles).

**Supplementary Material H**

In this set of simulations, we examine the influence of alternative explanations for changes in the speed and accuracy of responses. These reflect the assumptions of the Fast guess (Ollman, 1996; 1970) and deadline (Yellot, 1971) models respectively.

Both models assume that performance reflects a mixture of trials in which participant have engaged in a stimulus-controlled response, and a proportion of guess responses. For our simulations, we use the DDM to simulate trials from the control process. Note that our results do not depend on the choice of implementation for the controlled-processing responses; it is the effect of increasing the proportion of guess responses that is relevant for our purposes. The results of these simulations are shown in Figure H1. We reproduce our original DDM plots in the first row for comparison. We fix the parameters to be the same as those in the third row of Table G1 for all individuals, omitting starting point variability.

**Fast guess model (2^nd^ row)**

In the fast guess model, it is assumed that participants guess before engaging in processing the stimulus, therefor guess responses chance accuracy and a mean RT that is faster than their controlled-processing responses. We make a simplifying assumption here that the RTs for fast guesses are drawn from a normal distribution (M = 250ms, SD=20ms) for all participants. We vary the proportion of fast guesses from 0 to .3 in increments of .05. Increasing the proportion of fast guesses does not greatly impact upon RT costs and error costs (note the variation in the third column of the second row compared to the panel above it). A higher proportion of guesses responses slightly reduces both the RT cost and error cost, leading to a positive correlation. This is because an equal number of errors with equivalent RTs are added to both conditions, which slightly reduces the influence of controlled stimulus processing. Taken to the extreme, an individual who guesses on every trial would have and RT cost of 0ms and an error cost of 0% (chance accuracy).

**Deadline model (3^rd^ row)**

To simulate the impact of a self-imposed deadline, we make the assumption that participants engage in controlled-processing on every trial, but they will guess if a decision has not been reached before a specified duration. We vary the deadline on decision time between 200ms and 500ms in intervals of 50ms. Again, we make the simplifying assumption that guess responses are draw from a normal distribution with a mean equivalent to the deadline, and a standard deviation of 20ms.

The impact of decreasing the deadline is similar to that of reducing response caution in our main simulations – variation in response deadlines leads to a negative correlation between RT costs and error costs. This is because guesses are more likely to be made in the difficult condition, increasing the number of errors and thus the error costs. At the same time, the RT cost is reduced because the deadline does not allow for slow responses in the difficult condition, which are necessary for large RT costs.


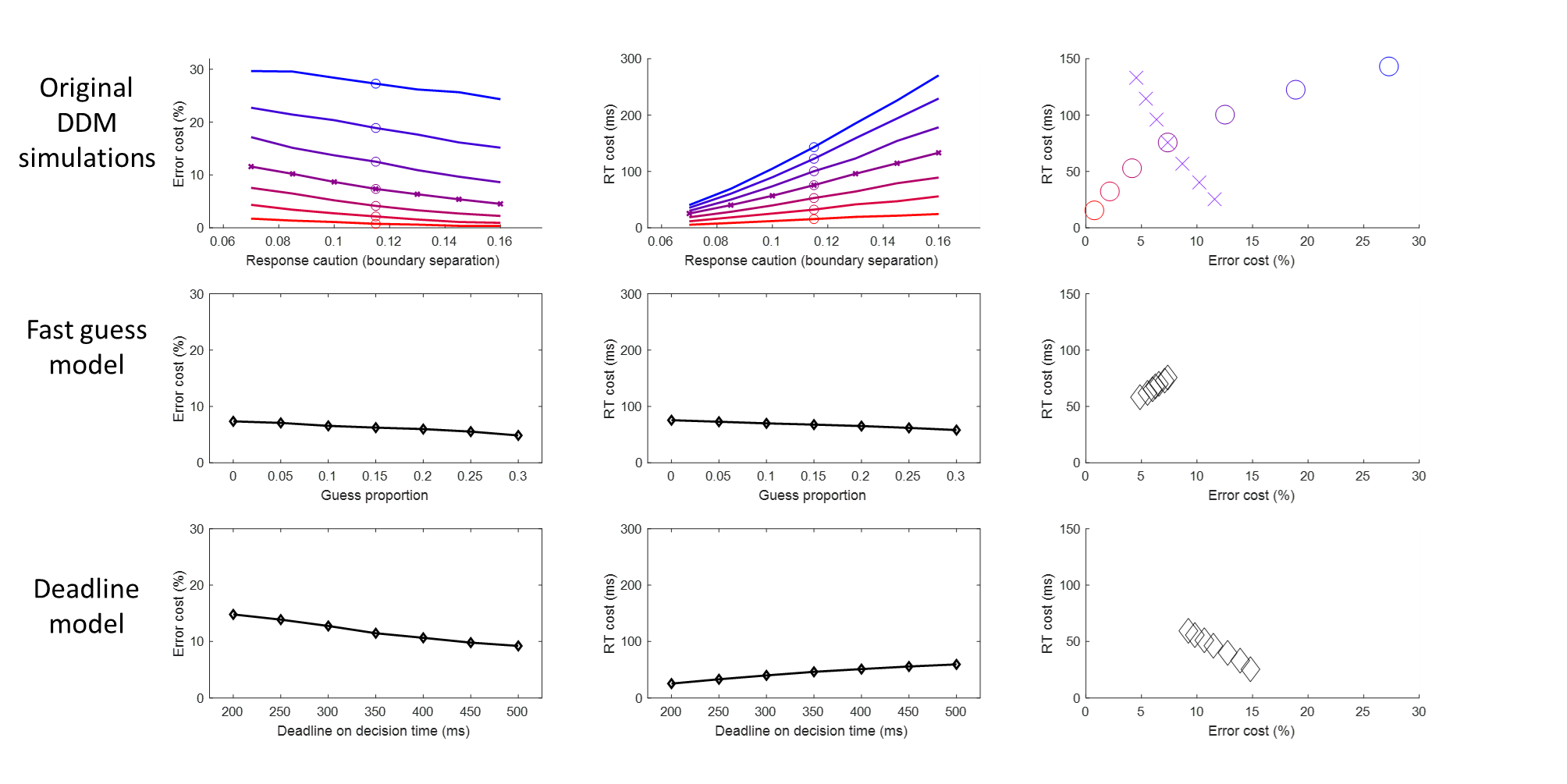


Figure H1. Simulated error costs and RT costs produced by drift-diffusion model, in combination with alternative sources of behaviour. The top row reproduces the results from our DDM simulations in the main text (See Figure 6). Variation in response caution is shown on the x-axis. Variation in response selection (the difference in drift rates in congruent and incongruent conditions) is indicated by different lines, ranging from low selection ability (blue) to high selection ability (red). The first and second columns show the patterns of error costs and RT costs, respectively, as a function of variation in both caution and response selection as implemented in the different models (see main text for details). The third column shows the correlation between RT costs and error costs that arise from holding response selection constant and allowing caution to vary (purple line and crosses), and for allowing response selection to vary while caution is held constant (grey line and circles). The second and thirds rows show the effect on RT costs and error costs where response selection and response caution are held constant, and the proportion of fast guesses is increased (second row) or an internal response deadline is decreased (third row).

**Supplementary Material I**

In this section, we provide simulations corresponding to Prediction 1 in Part 3 of the main text, where we predict that the correlation between RT costs and error costs will be more positive under instructions to emphasises speed relative to accuracy. We make this prediction on the assumption that speed instructions prompt individuals to reduce, and become more homogenous in, their levels of response caution. Here, we provide simulations illustrating this effect, as well as simulation the predictions of a fast guess and deadline account. As in the previous section, we use the DDM to simulate controlled processing trials in the fast guess and deadline model scenarios.

In each of the three scenarios (boundary change, fast guess, deadline), we simulated 5000 individuals performing a choice RT task with two conditions (e.g. congruent, incongruent) under speed and accuracy emphasis. Simulations consisted of 5000 trials per condition. The parameters used are shown in Table I1. To reflect the observation that mean RTs and accuracy rates are correlated within individuals, we imposed a correlation of r=.8 when generating drift rates for congruent and incongruent trials. Likewise, in the scenario in which the SAT was accounted for by a change in boundary separation, we imposed a correlation of r=.8 on boundary separation values under accuracy and speed emphasis. In the fast guess scenario, we made the simplifying assumption that guess RTs were taken from a normal distribution common to all individuals (M=250ms, SD=20ms). In the deadline scenario, we assumed guess response RTs were taken from a normal distribution with a mean deadline for that individual (SD=20ms).

|  | Boundary - accuracy emphasis | Boundary - speed emphasis | Drift rate congruent | Drift rate incongruent | non-decision time | Proportion guess | Deadline |
| --- | --- | --- | --- | --- | --- | --- | --- |
| Boundary change | .12 (.03) | .07 (.015) | 0.45 (.07) | 0.3 (.07) | 300ms | - | - |
| Fast guess | .12 (.03) | | 0.45 (.07) | 0.3 (.07) | 300ms | .2 (.05) |  |
| Deadline | .12 (.03) | | 0.45 (.07) | 0.3 (.07) | 300ms | - | 540ms (20) |

Table I1. Average parameters for the drift-diffusion model used in our simulations of the correlation between RT costs and error costs under speed-emphasis and accuracy emphasis. Standard deviations (across individuals) are shown in parentheses.

Note. Fast guess responses took an RT generated from a normal distribution (M=250ms, SD=20ms) with chance accuracy. In the deadline model, (late) guesses took an RT generated from a normal distribution with a mean corresponding to the individual’s deadline (SD=20ms).

The results of these simulations are shown in Figure I1. An increase in the correlation between RT costs and error costs was observed under a speed emphasis in both the boundary change and deadline scenarios. Our observed results (Table 3 in the main text) are therefore compatible with both of these accounts. A decrease in the RT cost – error cost correlation was observed in the fast guess scenario. See main text for further discussion. The scatter plots of our observed data are shown in Figure I2


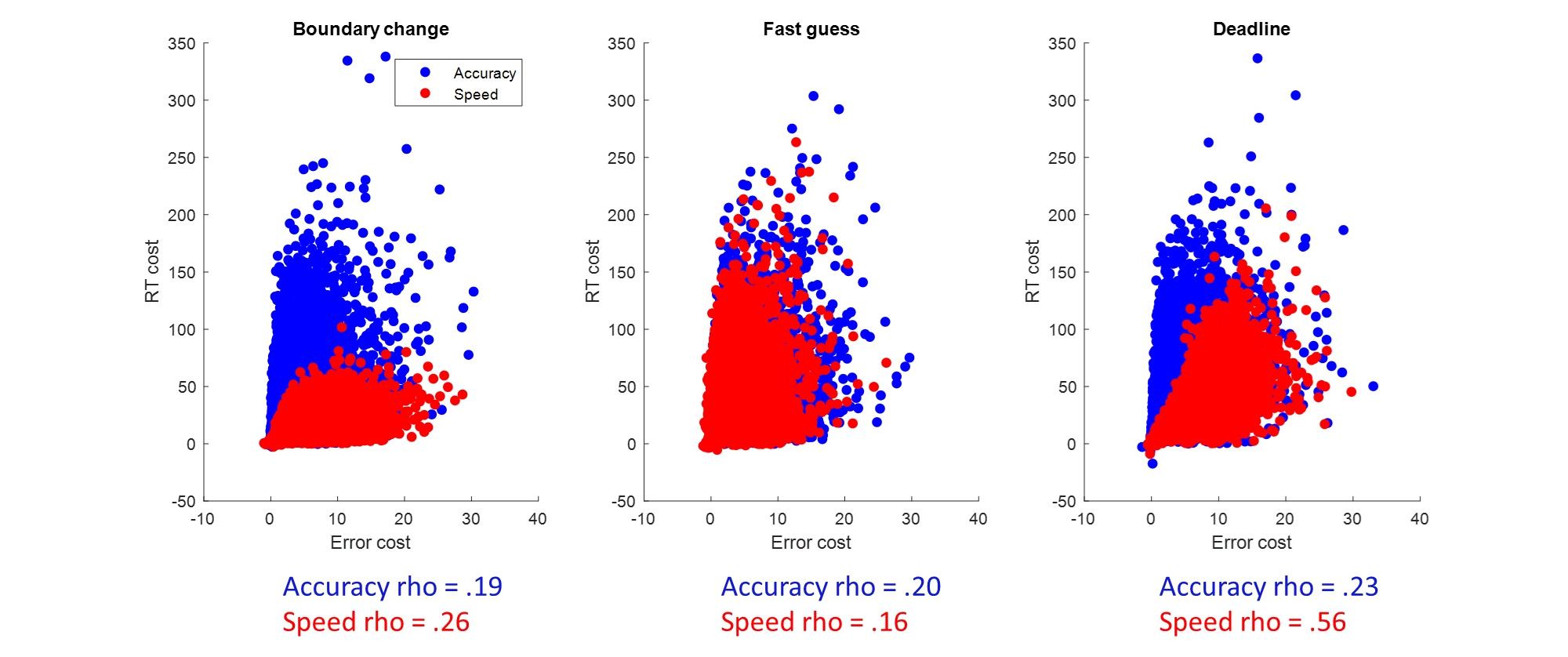
Figure I1. Correlations between RT costs and error costs under speed emphasis (red) and accuracy emphasis (blue) in three scenarios. The first (left) scenario reflects the assumption made in the main text that under speed emphasis individuals lower, and become more homogenous in, their response boundaries. The second (middle) scenario assumes that individuals do not lower their boundary under speed emphasis, instead making a higher proportion of fast guess responses. The third (middle) scenario assumes that individuals guess if a controlled-stimulus response has not occurred before some internally generated deadline. The more positive correlations we observe under speed emphasis in our data are compatible with the first and third scenarios, but not with a fast guess account.

Figure I2. Correlations between RT costs and error costs under speed emphasis (red) and standard instructions (to be both fast and accurate; blue) observed in six datasets (see Table 3 in main text).
